# RNA sequencing depth guidelines for the study of alternative splicing

**DOI:** 10.1093/nar/gkag653

**Published:** 2026-11-11

**Authors:** Zoe Chervontseva, Olga Tsoy, Lena Maria Hackl, Sabine Ameling, Sören Franzenburg, Markus Hoffmann, Lina Liv-Willuth, Hye Kyung Lee, Ludwig Knabl, Priscilla A Furth, Uwe Völker, Lothar Hennighausen, Jan Baumbach, Tim Kacprowski, Markus List

**Affiliations:** Institute for Computational Systems Biomedicine, University of Hamburg, Hamburg 22607, Germany; Institute for Computational Systems Biomedicine, University of Hamburg, Hamburg 22607, Germany; Computer Science Department, Vrije Universiteit Amsterdam, Amsterdam 1081 HV, The Netherlands; Institute for Computational Systems Biomedicine, University of Hamburg, Hamburg 22607, Germany; Interfaculty Institute for Genetics and Functional Genomics, University Medicine Greifswald, Greifswald 17475, Germany; German Centre for Cardiovascular Research (DZHK), Partner Site North, Greifswald 17475, Germany; Institute of Clinical Molecular Biology, Kiel University, Kiel 24105, Germany; Department of Biochemistry and Molecular & Cellular Biology, Georgetown University Medical Center, Washington, DC 20007, United States; Data Science in Systems Biology, TUM School of Life Sciences, Technical University of Munich, Freising 85354, Germany; National Institute of Diabetes, Digestive and Kidney Diseases, National Institutes of Health, Bethesda, MD 20892, United States; Klinische Mikrobiologie und Hygiene, TyrolPath Obrist Brunhuber GmbH, Tyrol, Austria; Data Science in Systems Biology, TUM School of Life Sciences, Technical University of Munich, Freising 85354, Germany; Georgetown University, Washington, DC 20057, United States; Interfaculty Institute for Genetics and Functional Genomics, University Medicine Greifswald, Greifswald 17475, Germany; German Centre for Cardiovascular Research (DZHK), Partner Site North, Greifswald 17475, Germany; National Institute of Diabetes, Digestive and Kidney Diseases, National Institutes of Health, Bethesda, MD 20892, United States; Institute for Computational Systems Biomedicine, University of Hamburg, Hamburg 22607, Germany; Institute of Mathematics and Computer Science, University of Southern Denmark, Odense 5000, Denmark; Institute of Data Science in Biomedicine, TU Braunschweig, Braunschweig 38106, Germany; Braunschweig Integrated Centre of Systems Biology, TU Braunschweig, Braunschweig 38106, Germany; Data Science in Systems Biology, TUM School of Life Sciences, Technical University of Munich, Freising 85354, Germany; Munich Data Science Institute, Technical University of Munich, Garching 85748, Germany

## Abstract

A key parameter in the experimental design of RNA-seq projects is the choice of sequencing depth. Considering a limited budget, one needs to find a trade-off between the number of samples and the sensitivity of the analysis, particularly concerning lowly expressed genes. While previous studies have proposed a lower bound for the comprehensive analysis of differential gene expression, for the analysis of alternative splicing, it has only been proposed for human adipose tissue. However, alternative splicing differs across tissues and conditions. We analyzed publicly available and newly generated deep-sequenced paired-end RNA-seq samples (between 150 and >500 million reads, read length 50–150 bp) from human buffy coat cells and diverse sets of tissues, including gluteal subcutaneous fat, heart, and hypothalamus. Our results show that the sequencing depth typically used in published cohorts is not sufficient to comprehensively capture the landscape of alternative splicing. This motivates the use of deeper sequencing or long-read technologies in future studies. Toward this goal, we offer guidelines and a website (https://sequencing-depth4splicing.com/) for choosing a suitable sequencing depth.

## Graphical Abstract

**Graphical Abstract:**
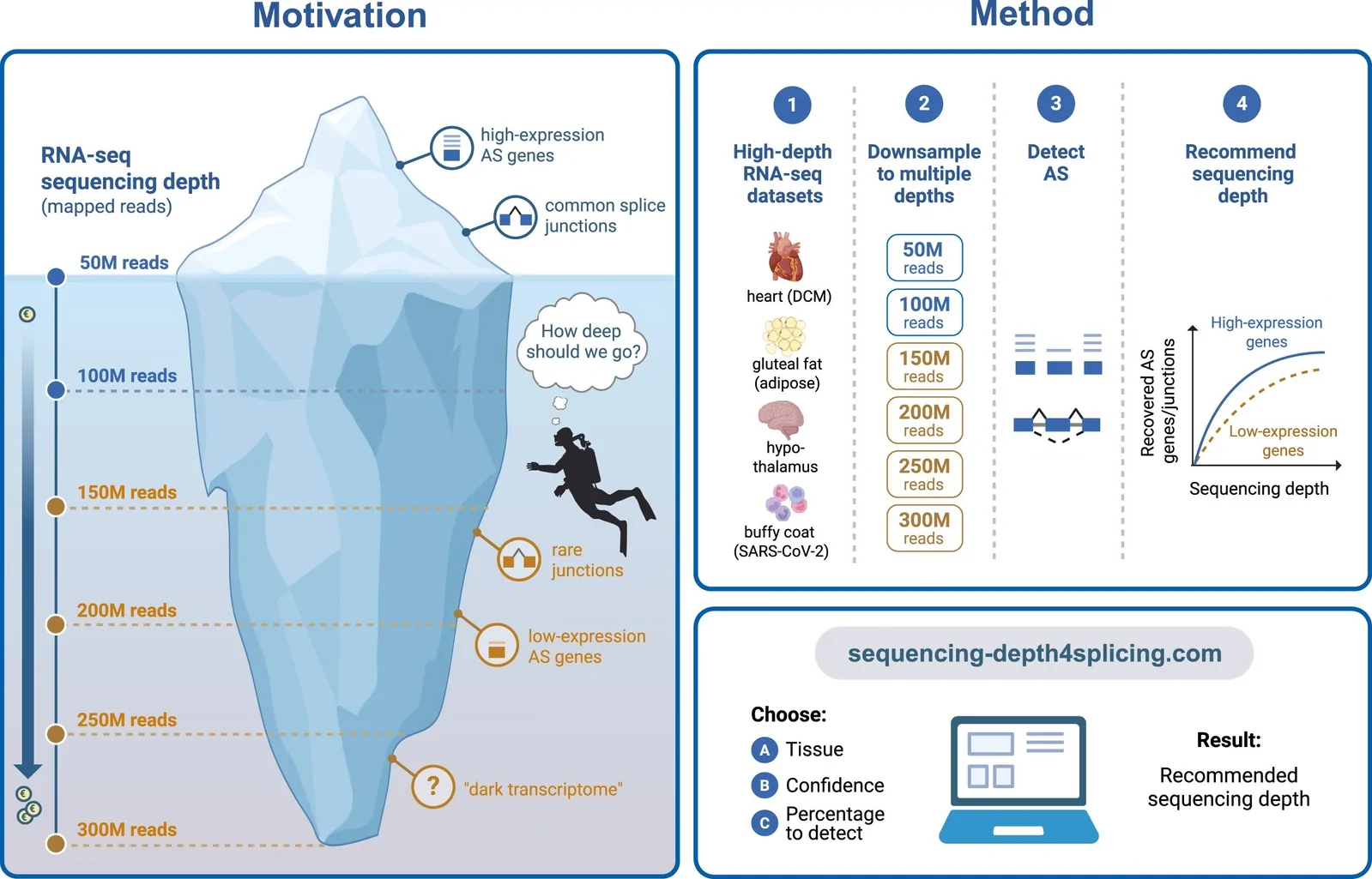

## Introduction

Short-read RNA sequencing is commonly used to study alternative splicing (AS) due to low sequencing error, the possibility of counting the abundance of sequences, and cost-effectiveness. Robust detection of AS events depends on the sequencing depth, which is especially crucial if a researcher aims to detect low-abundant transcripts [1]. Yet, it remains unclear how many reads are required for comprehensive coverage of AS in human samples. Considering the trade-off between sequencing depth and sample number, guidelines for the appropriate experimental design are needed.

The impact of sequencing depth on AS detection has been partially tackled in evaluating AS tools. SUPPA2 evaluation demonstrated that at 120 M reads, the proportion of true positive detections reaches almost 100% for exon skipping but 90% for alternative splice site events [2]. In the Cufflinks paper, the authors demonstrated that the recall suffered for low abundant transcripts even at 100 M reads [3]. When evaluating CASH [4], the authors demonstrated a notable increase in the performance of AS detection tools with the increase of sequencing depth from 50 to 100 M reads. We also partially addressed this question while evaluating the AS simulation tool ASimulatoR [5]. In the study, we simulated the dataset with 200 M reads. We observed the increase in AS detection performance between 50 and 100 M reads, as well as a steadily increasing number of detected AS events with a sequencing depth of >100 M reads. In our benchmark of AS detection tools [6], we observed that most AS detection tools demonstrate high precision regardless of the sequencing depth, but recall further increases even at 200 M reads.

However, all of the studies mentioned above use simulated data, where the number of AS events is chosen artificially and might not reach the complexity of the biological data. Moreover, most of the studies simulated <100 M reads (Supplementary Table S1). Finally, the primary focus of the AS detection tools evaluations is to demonstrate the performance and robustness of a tool. Thus, these studies do not answer the question of the RNA sequencing depth threshold for comprehensive AS detection.

Several studies addressed the sequencing depth threshold question for gene expression analysis. Estimates for the appropriate sequencing depth for differential gene expression analysis range from 200 to 300 M [7] reads [8] for the human genome. Bass *et al*. stated that 70% of all studies are “undersequenced” for thorough differential gene expression analysis [9]. The number of studies concerning AS is still limited. The SEQC Consortium investigated the discovery of exon–exon junctions and demonstrated that new junctions can still be detected even at 1 billion reads [10]. Lihuh *et al*. investigated the limitation of RNA-seq for detecting rare transcripts using the data from *Drosophila melanogaster* with spike-ins [11]. They estimated that at least 68 M uniquely aligned single-end 36-bp reads are needed to detect transcripts present at one copy per cell. Considering the larger size of the human genome and the larger number of genes, this number should be much larger for human data analysis. Hardwick *et al*. [12] presented an interesting approach that involves adding a synthetic transcriptome with known transcript sequences (sequencing spike-ins, “sequins”). They observed that even at 100 M reads, they could not detect the complete assembly. One study investigated sequencing-depth requirements in human adipose tissue and suggested a lower bound of 150 M paired reads [8]. More recently, Ladwig *et al*. analyzed how read depth and read length affect MAJIQ-based differential splicing analysis in controlled ENCODE knockdown datasets [13]. While this work highlights the importance of RNA-seq library characteristics for AS analysis, it does not address tissue-specific sequencing-depth requirements for comprehensive AS detection, particularly in lowly expressed genes. We analyzed RNA-seq data from diverse human tissues and conditions, considering published sources as well as very deeply sequenced heart tissue samples (>500 M reads). Since the sensitivity of AS detection depends on the expression level of a gene, we considered genes with low expression levels (TPM < 0.1, 0.1 ≤ TPM < 0.5, and 0.5 ≤ TPM < 1) to focus on low abundant transcripts, and high expression levels (1 ≤ TPM < 10, TPM ≥ 10) to suggest different lower bounds depending on a gene expression level.

## Materials and methods

The poly(A) RNA-seq datasets used in the study are listed in Table 1. RNA-seq data of human buffy coat cells isolated from whole blood from patients with SARS-CoV-2 variant Alpha (GSE190680 [14–17]) were obtained from Gene Expression Omnibus (GEO) [18]. This cohort contains 97 RNA-seq samples sequenced at various sequencing depths (from 50 to 150 M) and collected at different time points after the first symptoms [14]: 1–5, 10–14, and 15–30 days. RNA from the samples of the SARS-CoV-2 data had a quality RNA integrity (RIN) score between 8.8 and 10.0 with a mean of 9.82. The RIN value was measured by Agilent Bioanalyzer 2100.

**Table 1.** RNA-seq datasets used in the study

Dataset	Source	No. of samples	No. of paired reads (in M)	Read length (bp)
Human buffy coat white blood cells from SARS-CoV-2 infected patients	GSE190680	97	60–150	150
Adipose	GSE46323	2	455–519	101
Heart (DCM)	This study^a^	4	519–527	101
Hypothalamus	GTEx (V8)	102	10–236	76
Breast cancer (primary tissue)	TCGA	100	82–189	50

a Due to the restrictions of the patient consent, data can only be available upon request from University Medicine Greifswald (see the “Data availability” section).

Total RNA was extracted from human frozen endomyocardial biopsies using TRIzol (Thermo Fisher Scientific, Waltham, MA, USA), followed by DNA digestion purification steps using the RNAase-Free DNase I Kit and RNA Clean-Up and Concentration kit according to the manufacturer’s instructions (both Norgen Biotek Corp., Thorold, ON, Canada). The quality of the extracted RNA was assessed using the Tape Station 4200 (Agilent, Santa Clara, CA, USA). All four samples exhibited a mean RIN of 8.5. Subsequently, a PolyA library was prepared based on 500 ng total RNA per sample using the TruSeq stranded messenger RNA (mRNA) kit (Illumina, San Diego, CA, USA). For sequencing, 200 pM of the final library was loaded on the NovaSeq 6000 System, S1 FlowCell (2 × 100 bp paired-end reads). A sequencing depth of ~500 M clusters per sample was obtained and recorded at the Competence Centre for Genomic Analysis in Kiel.

RNA-seq samples from human adipose tissue before and after treatment (exposure to endotoxin) (GSE46323) were also downloaded from GEO. RNA-seq data from the hypothalamus (SRR1443713) have been downloaded from the Genotype-Tissue Expression (GTEx) project [19]. RNA-seq data from breast cancer (primary tissue) have been downloaded from the NIH National Cancer Institute GDC data portal https://portal.gdc.cancer.gov/projects/TCGA-BRCA (sample-IDs: https://doi.org/10.6084/m9.figshare.22361005.v1).

All RNA-seq data (except the data from TCGA) were obtained as raw reads and aligned to the human reference genome GRCh38.p13 with STAR 2.7 (default parameters, two-pass mode) [20] as this tool has been demonstrated to be the optimal tool for a human genome analysis [6, 21]. The TCGA data have been obtained as alignments in the BAM format. The gene counts were calculated by featureCounts from Subread 2.0 [22] and normalized to transcripts per million (TPM). Downsampling of aligned reads was performed with samtools 1.7 [23] using the subsampling option (“view -s”). The subsampling procedure in samtools randomly eliminates RNA-seq reads down to the requested amount. By design, the downsampling procedure of samtools takes into account only mapped reads. Thus, for all analyses in this study, we used the number of mapped reads as an indication of sequencing depth. The number of mapped reads of the analyzed samples has been obtained from the STAR log files or samtools flagstat output for the TCGA samples. The number of expressed junctions has been obtained using regtools 0.5.2 [24].

Among the published tools that can detect AS events in one condition, we chose MAJIQ [25], which showed high precision, acceptable recall, and good run time in a recently published benchmark [6]. MAJIQ also detects both known and novel AS events. We ran an AS analysis using MAJIQ 2.1 with default parameters. While MAJIQ 2.1 uses the notation of “local splicing variant”—alternative junctions bound to the same exon—and not AS event types (e.g. exon skipping, intron retention, etc.), we did not count AS events, but genes with AS and junctions involved in AS. Also, we considered only those “local splicing variants” with the percent splice-in (PSI) values >0.05 and <0.95 to decrease the number of false-positive AS event detections and focus on biologically relevant ones. The number of genes with AS and the number of junctions involved in AS were calculated and visualized by in-house Python scripts.

The gene- and transcript-related features have been calculated as follows. The compactness of a gene was defined as the total length of all exonic regions of a gene divided by its total length. The gene annotation was taken from gencode.v44. The list of heart-specific genes was taken from [26]. The regions of accelerated evolution were taken from [27]. The transcriptome-captured somatic mutations were identified from the RNA-seq reads with rnaseqmut (https://github.com/davidliwei/rnaseqmut).

We estimated the rarity of AS events by comparing to junctions detected in RNA-seq samples from the Sequence Read Archive compiled by intropolis v2 [28].

The Mann–Kendall trend test has been performed using the pymannkendall 1.4.3 Python package (https://pypi.org/project/pymannkendall/). The reported Mann–Kendall trend slope presents a magnitude of the monotonic trend. The GTEx Hypothalamus dataset lacks enough data points to run the trend test.

The functional enrichment for the genes with AS detected only at 200 M reads has been performed using g:Profiler [29]. The AS-aware enrichment was performed using NEASE [30] using the list of exon skipping events as an input. The events are visualized via the sashimi tool [31].

The webpage for the minimal sequencing depth estimation was deployed on netlify (https://sequencing-depth4splicing.com/).

The cost for detecting an additional gene with AS/alternative junction with an increasing number of mapped reads has been calculated according to the prices from the Competence Centre for Genome Analysis Kiel (CCGA Kiel, https://ccga.uni-kiel.de). We divided the cost of sequencing for each additional 50 M reads by the number of genes with AS/junctions identified with this new portion of reads.

All Python scripts used to generate the figures as well as the MAJIQ raw results and summary files are available at https://github.com/OlgaVT/RNA-seq_Depth_for_AS and https://doi.org/10.5281/zenodo.11655945.

## Results

### SARS-CoV-2 cohort

We investigated how the number of detected genes with AS events/junctions involved in AS events depends on a sequencing depth in real-world data. However, a sequencing depth >50–100 M reads is uncommon for large RNA-seq datasets. The SARS-CoV-2 cohort used in the study was chosen because of the deeper than usual amount of reads (up to 150 M) [14].

For each sample from the SARS-CoV-2 cohort, we calculated how many genes with AS events and how many junctions involved in AS events were detected. We considered genes with low expression (TPM < 0.1, 0.1 ≤ TPM < 0.5, and 0.5 ≤ TPM < 1) and high expression (1 ≤ TPM < 10, TPM ≥ 10), and AS events with 0.05 < PSI < 0.95. We examined how the number of detected genes/junctions depends on the number of uniquely mapped reads reported by STAR (Fig. 1 and Supplementary Fig. S1). The total number of genes and junctions in each category is shown in Supplementary Table S2. We tested if there was an increasing trend using the Mann–Kendall trend test and calculated the increase in the mean number of detections (Table 2). Percentage of new detections was calculated as the ratio between the mean value of detections at a certain sequencing depth and the mean total value of genes/junctions per expression category.

**Figure 1.**
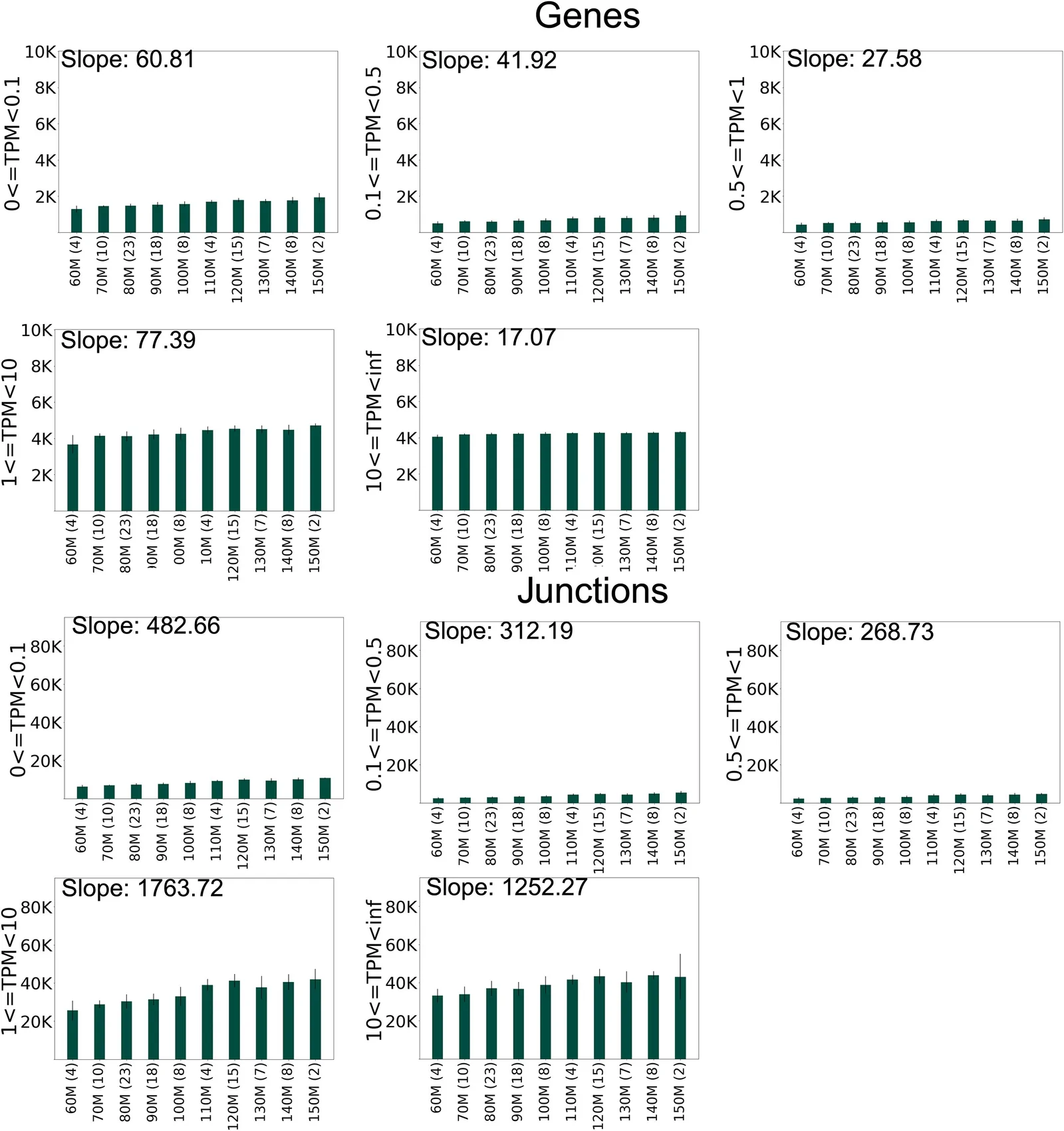
The number of genes with AS and junctions involved in AS detected from SARS-CoV-2 RNA-seq data with various numbers of mapped reads. The bar plots demonstrate the mean number of genes/junctions involved in AS. Slopes (a magnitude of the monotonic trend) are indicated for significant (*P*-value < .05) Mann–Kendall trend test results.

**Table 2.** The increase of the mean number and the percentage of genes and junctions involved in AS with increasing sequencing depth in the SARS-Cov-2 dataset

TPM	0–60 M	60–70	70–80	80–90	90–100	100–110	110–120	120–130	130–140	140–150	150 M+	Mean total number of genes/ junctions
Genes												
<0.1	1287.8 (20.9%)	+161.3 (+2.6%)	**+19.9** **(+0.3%)**	+63.4 (+1.0%)	**+25.4** **(+0.4%)**	+132.6 (+2.2%)	+91.2 (+1.5%)	**−56.7** **(−0.9%)**	**+43.9** **(+0.7%)**	+165.2 (+2.7%)	1932.5 (31.4%)	6149
[0.1, 0.5)	498 (11.8%)	+105.4 (+2.5%)	**−14.7** **(0.4%)**	+54.3 (+1.3%)	**+19.4** **(+0.5%)**	+107.4 (2.5%)	+48.1 (+1.1%)	**−19.6** **(−0.5%)**	**+6.9** **(+0.2%)**	+128.3 (+3%)	933.5 (22.2%)	4213
[0.5, 1)	416 (20.7%)	+104.7 (5.2%)	**−7.9** **(−0.4%)**	+29.5 (+1.5%)	**+10.8** **(+0.5%)**	+77.9 (+3.9%)	+25.7 (+1.3%)	**−13.4** **(−0.7%)**	**+3.2** **(+0.2%)**	+68.5 (+3.4%)	715 (35.6%)	2006
[1, 10)	3671.8 50.6%)	+473.4 +6.5%)	**−25.5** **(−0.4%)**	+97.5 (+1.3%)	**+27.3** **(+0.4%)**	+214.1 (+3%)	+77.2 (+1.1%)	**−31.4** **(−0.4%)**	**−30.2** **(−0.4%)**	+232.2 (+3.2%)	4706.5 (64.9%)	7254
≥10	4048.3 (79.2%)	+134.9 (+2.6%)	**+16.8** **(+0.3%)**	**+19.7** **(+0.4%)**	**+5.4** **(+0.1%)**	**+28.8** **(+0.6%)**	**+25.5** **(+0.5%)**	**−29.9** **(−0.6%)**	**+19.4** **(+0.4%)**	**+37.3** **(+0.7%)**	4306 (84.2%)	5112
Junctions												
<0.1	6283.3 (25.9%)	+716.1 (+2.9%)	+348.2 (+1.4%)	+383.6 (+1.6%)	+528.0 (+2.2%)	+1016.5 (+4.2%)	+673.7 (+2.8%)	−521.9 (−2.1%)	+686.4 (+2.8%)	+717.5 (+3.0%)	10 831.5 (44.6%)	24 302
[0.1, 0.5)	2407 (7.5%)	+429.4 (+1.3%)	**+90.3** **(+0.3%)**	**+304.4** **(+0.9%)**	**+272.1** **(+0.8%)**	+786.5 (+2.5%)	+458.5 (+1.4%)	−388.8 (−1.3%)	+440.0 (+1.4%)	+602.7 (+1.9%)	5402.5 (16.8%)	32 095
[0.5, 1)	2212.8 (10.1%)	+444.8 (+2.0%)	**+92.7** **(+0.4%)**	**+185.5** **(+0.9%)**	**+174.2** **(+0.8%)**	+948.25 (+4.3%)	+368.7 (+1.7%)	−418.1 (−1.9%)	+312.6 (+1.4%)	+415.0 (+1.9%)	4736.5 (21.7%)	21 823
[1, 10)	25 612.5 (17.7%)	+3267.7 (+2.3%)	+1491.1 (+1.0%)	**+1043.3** **(+0.7%)**	+1583.0 (+1.1%)	+5902.9 (4.1%)	+2393.7 (+1.7%)	−3573.6 (−2.5%)	+2731.9 (+1.9%)	+1544.5 (1.1%)	41 997 (29.0%)	144 588
≥10	31 105.5 (21.4%)	**767.3** **(+0.5%)**	+3083.7 (+2.0%)	**−321.2** **(−0.2%)**	+2191.5 (+1.4%)	+2685.5 (+1.7%)	+1760.2 (+1.1%)	−3023.5 (−2.0%)	+3586.8 (+2.3%)	**−830.3** **(−0.5%)**	43005.5 (27.8%)	154 833

The column “0–60 M” indicates the mean number and the percentage of genes/junctions detected in the samples with a sequencing depth of <60 M reads. The column “150 M+” indicates the mean number and the percentage of genes/junctions detected in the samples with the highest sequencing depth of >150 M reads. Bold text indicates an increase of <1% new detections.

The Mann–Kendall test confirmed the increasing trend for all expression levels—all calculated slopes are positive (Fig. 1). Table 2 shows that for almost all gene expression categories (except TPM >10), the increase from 60 to 150 M reads results in ~10%–15% new detections. Even an increase from 140 to 150 M still results in >1% of new detections. Thus, to detect AS events with 0.05 < PSI < 0.95 in the genes with TPM < 10, a sequencing depth of 150 M reads is insufficient. However, for very highly expressed genes (TPM >10), the threshold might be around 70 M reads.

The trend is similar for the number of detected junctions involved in AS (Table 2). For junctions in genes with TPM <10, the increase from 60 to 150 M results in ~12%–19% new detections. Also, the increase from 140 to 150 M reads still results in >1% new detections, confirming that 150 M reads are insufficient for covering genes with low expression in AS analysis. However, for junctions in very highly expressed genes, the increase declines around 110–120 M reads, indicating a much lower threshold for this gene expression category.

While we observe a stable positive trend, the values for the number of genes/junctions involved in AS events show considerable variation across samples. This motivated us to visually investigate and confirm for several genes that the RNA-seq data does support the expression of junctions that could be detected only in deeply sequenced samples and that the corresponding events do not merely represent false positive AS event detections (Supplementary Fig. S2).

### Deep-sequenced cohorts

After the analysis of the SARS-CoV-2 cohort, the question remains: how will the number of AS event detections increase with the sequencing depth of >150 M reads? We searched for datasets with >150 M reads and discovered two published datasets with paired-end mRNA RNA-seq samples: two samples from the publicly available RNA-seq dataset from the human adipose tissue and one hypothalamus RNA-seq sample from the GTEx cohort. We additionally deeply sequenced four samples from the heart tissues of patients suffering from dilated cardiomyopathy (DCM).

We examined how the robustness and sensitivity of AS detection change with an increasing number of mapped reads (Fig. 2). We downsampled each sample in the adipose and heart (DCM) datasets 100 times to 300, 250, 200, 150, 100, and 50 M reads (Fig. 3), and hypothalamus dataset to 200, 150, 100, and 50 M (Supplementary Fig. S3). As before, we divided the detected genes into groups according to the expression level: low expression levels (TPM < 0.1, 0.1 ≤ TPM < 0.5, and 0.5 ≤ TPM < 1), and high expression levels (1 ≤ TPM < 10, TPM ≥ 10). Then, we counted the number of detected genes/junctions involved in AS with 0.05 < PSI < 0.95. The total number of genes in each category is shown in Supplementary Table S2. In line with the SARS- CoV-2 dataset analysis, we applied the Mann–Kendall Trend test to detect the significance of the trend and the sign of a slope and calculated the increase in the number of detections (Table 3 and Supplementary Tables S4–S6).

**Figure 2.**
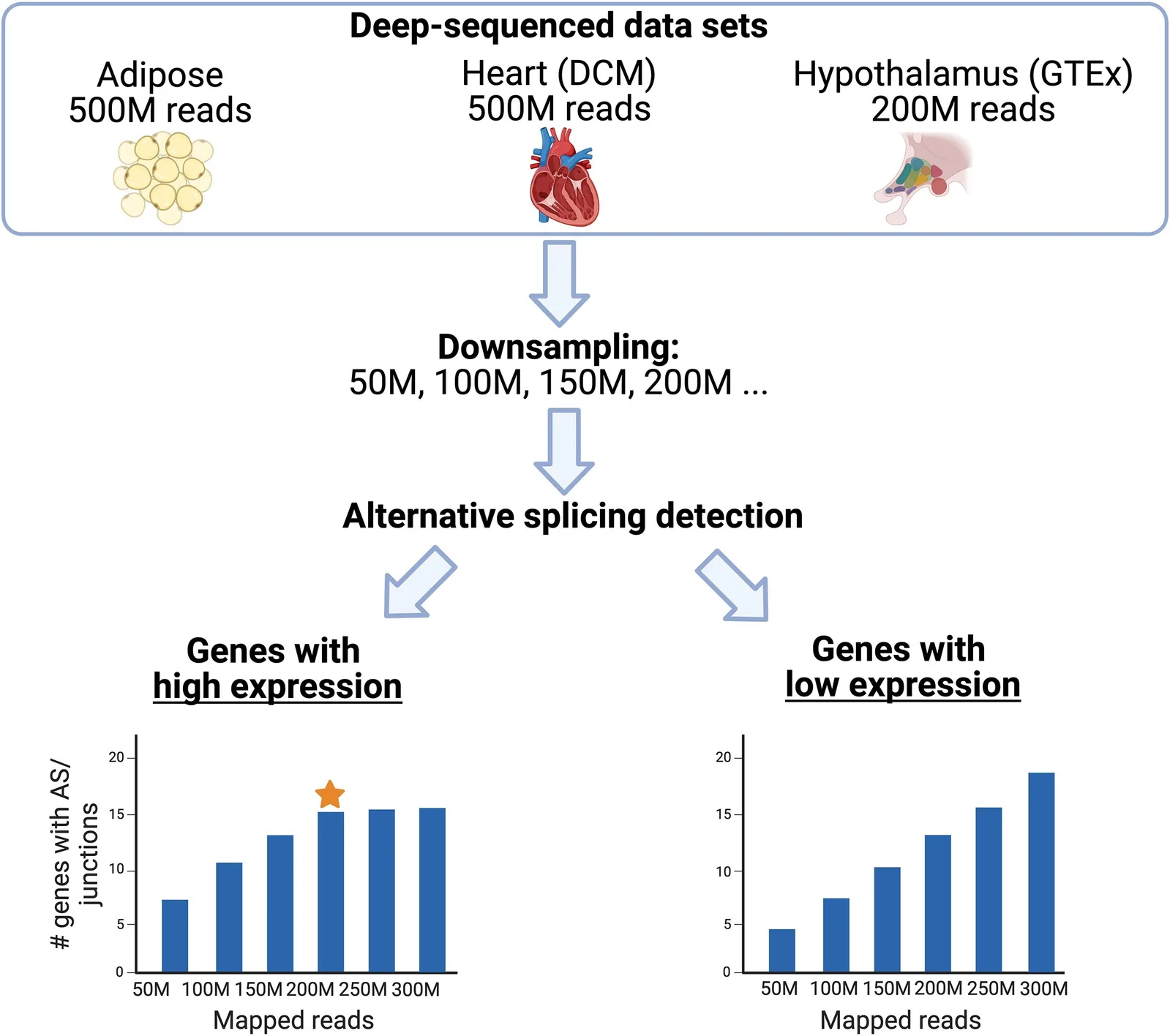
The strategy to estimate the number of mapped reads needed for AS analysis from three deep-sequenced datasets. We expect that new AS events will be steadily detected for genes with low expression due to an increase in sensitivity. In contrast, for highly expressed genes, a saturation effect is expected. The star marks the threshold for the sequencing depth we aim to identify. Note that the bars shown here are only meant for illustration and do not represent the results. Created in BioRender. List, M. (2026) https://BioRender.com/zqwh68l.

**Figure 3.**
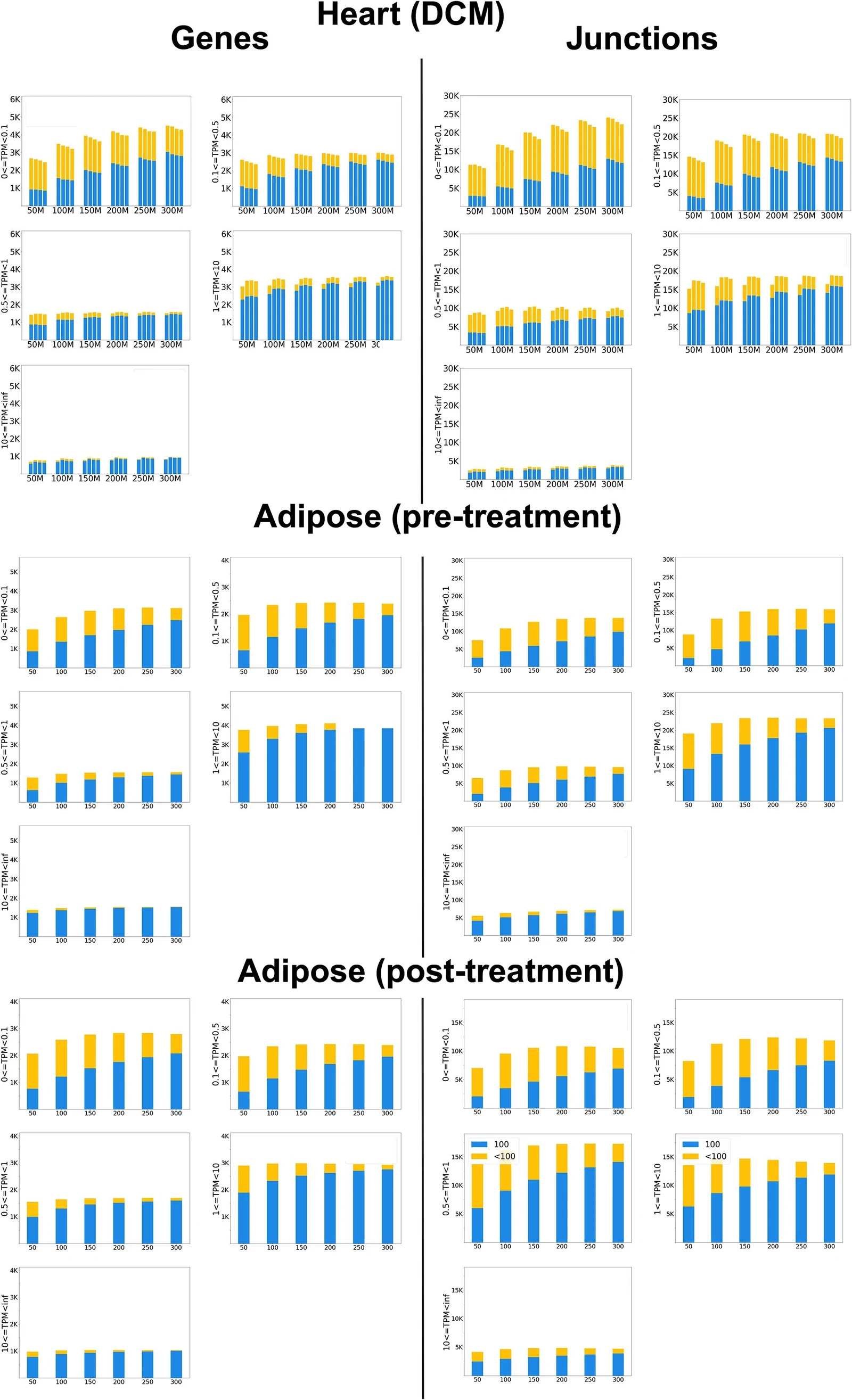
The number of genes and junctions involved in AS detected in 100 (blue) and <100 (yellow) out of 100 subsamples. Together, blue and yellow bars represent the number of genes and junctions involved in AS detected in at least one subsample. Each stacked bar depicts one sample. The slopes for significant (*P*-value < .05) results of the Mann–Kendall trend test are shown in Supplementary Table S3.

**Table 3.** The increase of the mean number and the percentage of genes and junctions involved in AS events with increasing sequencing depth in the heart (DCM) dataset

TPM	50 M	50–100	100–150	150–200	200–250	250–300	300 M	Mean total number of genes/junctions
Genes (in at least one subsample)								
<0.1	2571.3 (19.1%)	+779.3 (+5.8%)	+435.8 (+3.2%)	+271.8 (+2.0%)	+215 (+1.6%)	**+119** **(+0.9%)**	4292 (32.7%)	13 436
[0.1, 0.5)	2483.5 (50.8%)	+279.25 (+5.7%)	+111 (+2.3%)	+47 (+1.0%)	**+16.5** **(+0.3%)**	**+17** **(+0.3%)**	2954.3 (60.4%)	4889
[0.5, 1)	1453.3 (66.4%)	+65 (+3.0%)	**+16.5** **(+0.8%)**	**+8.8** **(+0.4%)**	**+5.3** **(+0.2%)**	**+4** **(+0.2%)**	1552.8 (71%)	2187
[1, 10)	3267 (65.8%)	+78.5 (+1.6%)	**+43.5** **(+0.9%)**	**+41** **(+0.8%)**	**+36.8** **(+0.7%)**	**+23.5** **(+0.5%)**	3490.3 70.3%)	4967
≥10	752.8 (57.6%)	+75.5 (+5.8%)	+36 (+2.8%)	+27.8 (+2.1%)	+18.8 (+1.4%)	+15.8 (+1.2%)	926.5 (70.9%)	1307
Genes (in 100 subsamples)								
<0.1	891 (6.6%)	+588.3 (+4.4%)	+441.3 (+3.3%)	+381 (+2.8%)	+301 (+2.2%)	+291.8 (+2.2%)	18 954.3 (21.5%)	13 436
[0.1, 0.5)	1014.3 (20.7%)	+680.5 (+13.9%)	+348.3 (+7.1%)	+223 (+4.6%)	+142.5 (+2.9%)	+118 (+2.4%)	2526.5 (51.7%)	4889
[0.5, 1)	856.8 (39.2%)	+281.8 (+12.9%)	+127.3 (+5.8%)	+80.3 (+3.7%)	+48.3 (+2.2%)	+41.8 (+1.9%)	1436 (65.7%)	2187
[1, 10)	2415.8 (48.6%)	+400 (+8.1%)	+180.5 (+3.6%)	+121.3 (+2.4%)	+100.5 (+2.0%)	+72.3 (+1.5%)	3290.3 (66.2%)	4967
≥10	631.5 (48.3%)	+94.3 (+7.2%)	+55.8 (+4.3%)	+43.8 (+3.3%)	+33 (+2.5%)	+29.5 (+2.3%)	887.8 (67.9%)	1307
Junctions (in at least one subsample)								
<0.1	10 930 (10.2%)	5206.5 (+4.8%)	3138.8 (+2.9%)	1911.8 (+1.8%)	1293.5 (+1.2%)	**735.3** **(+0.7%)**	23 215.8 (21.6%)	107 673
[0.1, 0.5)	13 584.5 (14.6%)	4321.8 (+4.5%)	1578.8 (+1.7%)	**494.8** **(+0.5%)**	**82.8** **(+0.09%)**	**−49.3** **(−0.05%)**	20283.3 (21.3%)	95 009
[0.5, 1)	8416 (15.9%)	+1332 (+2.5%)	**+154.8** **(+0.3%)**	**−99.8** **(−0.2%)**	**−112.8** **(−0.2%)**	**−96.5** **(−0.2%)**	9593.8 (18.2%)	52 799
[1, 10)	16 672 (13.9%)	**902.3** **(+0.8%)**	**232.3** **(+0.2%)**	**120** **(+0.1%)**	**89.3** **(+0.07%)**	**119.5** **(+0.1%)**	18 135.3 (15.1%)	120 282
≥10	2649.3 (8.9%)	+336.3 (+1.1%)	**+224.5** **(+0.8%)**	**+160.8** **(+0.5%)**	**+127.3** **(+0.4%)**	**+104.8** **(+0.4%)**	3602.8 (12.1%)	29 891
Junctions (in 100 subsamples)								
<0.1	2799 (2.6%)	+2318.8 (+2.2%)	+2017.8 (+1.9%)	+1851 (+1.7%)	+1671 (+1.6%)	+1622.3 (+1.5%)	12 279.8 (11.4%)	107 673
[0.1, 0.5)	3620.3 (3.8%)	+3466 (+3.6%)	+2290 (+2.4%)	+1724.5 (+1.8%)	+1463.5 (+1.5%)	+1197.5 (+1.3%)	13 761.8 (14.5%)	95 009
[0.5, 1)	3272.8 (6.2%)	+1748.5 (+3.3%)	+920.3 (+1.7%)	+645.3 (+1.2%)	+511.8 (+1.0%)	**+446.8** **(+0.8%)**	7545.3 (14.3%)	52 799
[1, 10)	9208.3 (7.7%)	+2407.3 (+2.0%)	+1278.3 (+1.1%)	**+973.3** **(+0.8%)**	**+825** **(+0.7%)**	**+699** **(+0.6%)**	15 391 (12.8%)	120 282
≥10	1979 (6.6%)	+362 (+1.2%)	**+263.5** **(+0.9%)**	**+211.3** **(+0.7%)**	**+183.3** **(+0.6%)**	**+170** **(+0.6%)**	3189 (10.6%)	29 891

Column “50 M” indicates the mean number and the percentage of genes/junctions detected in the samples with a sequencing depth of <50 M reads. The column “300 M” indicates the mean number and the percentage of genes/junctions detected in the samples with the highest sequencing depth of 300 M reads. Bold text indicates an increase of <1%.

We observed differences in how the number of genes with AS/junctions involved in AS changes with an increasing sequencing depth between genes/junctions. Specifically, we compared AS changes consistently detected in all 100 subsamples (Fig. 3, blue bar plots) to changes observed in at least one subsample (Fig. 3, blue + yellow bar plots). For genes/junctions detected consistently in all 100 subsamples, the Mann–Kendall trend test reported a significantly increasing trend for all datasets (Supplementary Table S3, “in 100 subsamples”). However, for genes/junctions detected in at least one subsample (Supplementary Table S3, “in at least one subsample”), the trend is not significant in the adipose (pretreatment) dataset for lowly expressed (0.5 ≤ TPM < 1) genes and for junctions from lowly expressed (0.5 ≤ TPM < 1) and highly expressed (1 ≤ TPM < 10) genes. The trend is also not significant in the adipose (posttreatment) dataset for lowly expressed (TPM < 0.1, 0.1 ≤ TPM < 0.5, and 0.5 ≤ TPM < 1) and highly expressed (1 ≤ TPM < 10) genes with AS and for junctions involved in AS from all gene expression levels except 0.5 ≤ TPM < 1.

Next, for most datasets, for genes with AS/junctions involved in AS found in 100 subsamples (Table 3 and Supplementary Tables S4–S6; “In 100 subsamples”), we observe >1% of new detections of genes/junctions even with the increase of sequencing depth from 250 to 300 M reads. The only exception is the number of junctions involved in AS from the Heart (DCM) dataset [Table 3, “Junctions (in 100 subsamples)”]. Thus, with higher sequencing depth, more AS events can be robustly found in the dataset. However, to confirm all AS events, a sequencing depth >300 M is needed.

On the other hand, the increase of genes with AS/junctions involved in AS for those found in at least one subsample slows down—above a certain sequencing depth, there are <1% of new detections (Table 3 and Supplementary Tables S4–S6, “In at least one subsample”). In this case, we suggest the threshold of 150–200 M reads for genes/junctions in the low expression category (TPM < 0.1, 0.1 ≤ TPM < 0.5, and 0.5 ≤ TPM < 1) and of 100–150 M reads for genes/junctions in the high expression category (1 ≤ TPM < 10, TPM ≥ 10).

### Impact of gene and AS event features on AS detection with increasing sequencing depth

To assess whether the additional splice junctions detected at higher sequencing depths are enriched for specific gene or junction properties, we analyzed the composition of robustly detected junctions involved in AS across downsampled DCM datasets from 50 to 300 M reads. For this analysis, robust detection was defined as detection in all 100 downsampling iterations at a given sequencing depth, thereby minimizing stochastic effects of subsampling. For each feature, we regressed the proportional representation of each category among detected junctions against sequencing depth and tested whether the most divergent feature categories showed significantly different coverage-dependent trends. Most features showed significant changes in proportional representation with increasing depth, with the exception of PSI and GC content (Fig. 4A). However, the absolute effect sizes were modest. The largest compositional shift was observed for gene expression: with increasing depth, the proportion of detected junctions from lowly expressed genes increased, whereas the proportions from medium-expressed genes and highly expressed genes decreased (Fig. 4B). This negative trend for highly expressed genes does not indicate reduced detectability; rather, it reflects that junctions in highly expressed genes are already captured at lower depth, while additional sequencing preferentially recovers junctions from lowly expressed genes. Similar compositional effects explain the decreasing proportion of protein-coding genes and CDS-localized junctions, together with modest increases in junctions from noncoding gene categories and untranslated regions.

**Figure 4.**
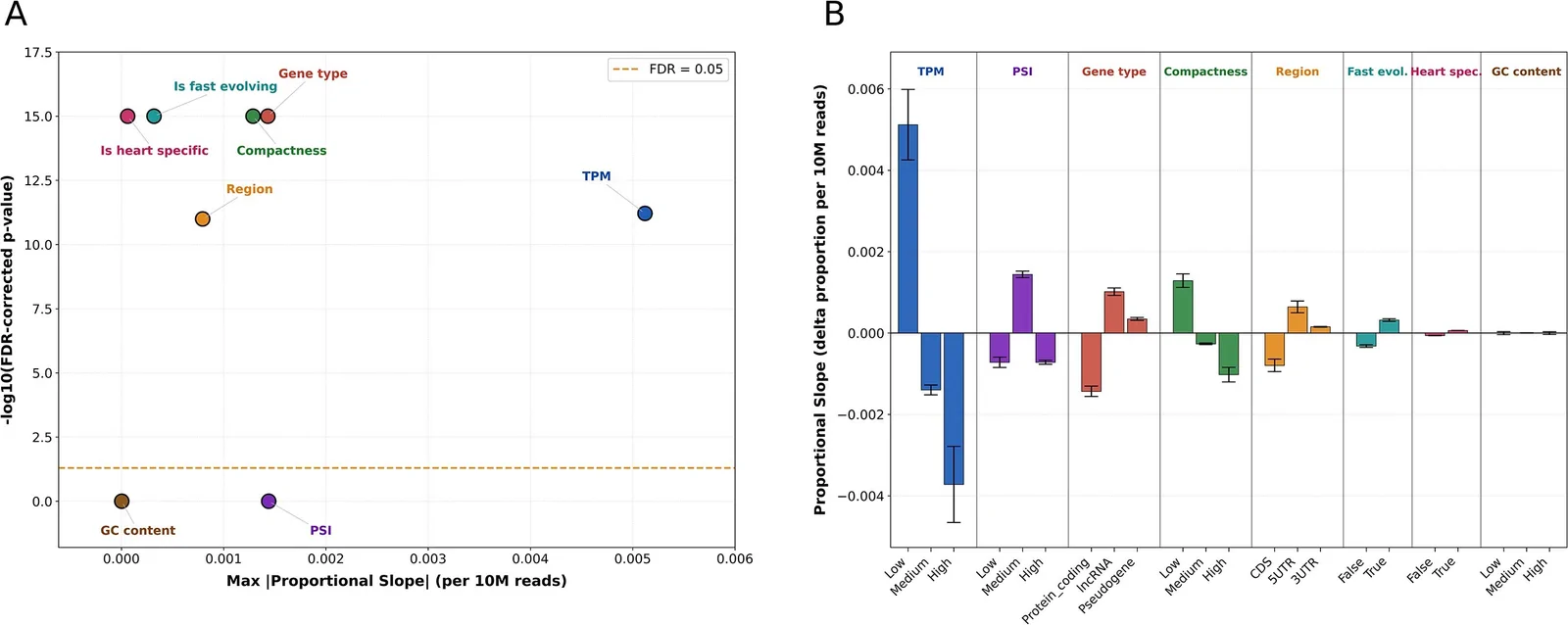
Impact of gene and junction features on the composition of detected splicing junctions across sequencing coverage. (**A**) Significance versus effect size for eight features of genes and junctions involved in AS. For each feature, the proportional representation of each subcategory among all detected junctions was regressed against sequencing coverage (50–300 M reads) using linear regression. The *x*-axis shows the maximum absolute proportional slope across subcategories (per 10 M reads); the *y*-axis shows—log_10_ of the BH-FDR-corrected *P-*value. The *P*-value per feature tests whether the most divergent pair of subcategories (high versus low for continuous; max-slope versus min-slope for categorical) differ significantly in their coverage-dependent trends, using a two-tailed *z*-test on the difference between their regression slopes. *P-*values are corrected across all eight features using the Benjamini–Hochberg procedure. The dashed line marks FDR = 0.05. (**B**) Proportional slopes (±1 SE) for each subcategory, grouped by feature and ordered by decreasing maximum |slope|. Continuous features (PSI, GC content, compactness, TPM) are divided into tertiles (low/medium/high); categorical features use up to three most abundant categories (two for binary features: fast-evolving status, heart-specific status). Data: splicing junctions detected in all 100 downsampling iterations.

Because several features were correlated with each other, including gene expression with compactness and GC content (Supplementary Fig. S4), we further fitted generalized estimating equation logistic models to estimate the joint association of all features with splice junction detectability while accounting for repeated measurements of the same junction across samples. In this multivariable analysis, gene expression remained by far the strongest predictor of detection probability across all sequencing depths (Supplementary Fig. S5). Other features had substantially smaller effects: PSI showed a modest positive association with detection, GC content showed a weak negative association, compactness remained close to neutral, and gene type, genomic region, fast-evolving status, and heart-specific status showed smaller and more depth-dependent effects. Thus, although gene and junction features influence which classes of AS junctions are progressively added with increasing sequencing depth, their effects are much smaller than the effect of expression and do not alter the main conclusion that increased coverage primarily improves AS detection by recovering junctions from low-abundance genes.

### Sequencing depth and rare AS events

Rare AS events provide isoforms with specialized functions or serve as a biomarker of a pathological condition. Increasing sequencing depth might facilitate the detection of such events. Rare AS events are usually defined as rarely detected in a cohort, however, the deep-sequencing cohorts present a limited number of samples. Thus, we used the following definitions of rare AS events: (i) AS events that rarely present across different tissues/conditions in publicly available datasets; and (ii) AS events that use noncanonical splice sites. We observed (Supplementary Fig. S6) that the proportion of junctions involved in AS and not found in any publicly available datasets from SRA tends to increase with the sequencing depth. The proportion of junctions with noncanonical splice sites steadily increases for most of the datasets (except the samples from adipose) (Supplementary Fig. S7). Overall, our findings might indicate that higher sequencing depth facilitates the detection of rare AS events.

### The influence of biological heterogeneity on the AS detection

Similarly to gene expression detection [32], AS detection might also be affected by biological variation. To assess the magnitude of the effect, we used the amount of transcriptome-captured somatic mutations identified from the RNA-seq reads (see the “Materials and methods” section) as a proxy of biological heterogeneity on a sample level. Our assumption is that samples with more heterogeneous cell populations (or more accumulated cell-intrinsic differences) should carry a higher number of unique variants detectable in RNA reads.

We observed that the average number of detected mutations across all analyzed samples increases approximately linearly with sequencing coverage (Supplementary Fig. S8, left), indicating that the estimate of biological heterogeneity is itself strongly coverage dependent. However, when plotted against the average number of mutations, the number of detected splice junctions reaches a plateau: beyond the coverage effect, additional heterogeneity in our samples only slightly contributes to the amount of junctions we can detect (Supplementary Fig. S8, right).

Taken together, these observations indicate that AS events detection is driven primarily by sequencing depth. Any additional contribution from biological heterogeneity, while plausible conceptually, has much smaller impact and is not the dominant factor for AS detection.

### The biological relevance of AS events found at 200 M reads

We investigated the biological relevance of the AS events found only at 200 M reads using various approaches. First, we extracted genes with AS detected in all 100 samples exclusively at 200 M reads but not in 50, 100, and 150 M, and performed functional gene enrichment with g:Profiler [29]. AS events unique to 200 M reads in highly expressed genes (TPM > 1) in the adipose sample after endotoxin treatment are enriched in the category “response to organic substance” (Supplementary Fig. S9), indicating that higher sequencing depth might reveal additional information about the response mechanism to endotoxin.

One of the datasets presents a disease phenotype—DCM. We extracted from the DisGeNET database [33] the list of genes associated with cardiomyopathies and overlapped it with the list of genes with AS events found only at 200 M. We found two low-expressed (TPM < 1) genes with AS that might be associated with cardiomyopathies: FKBP1B (DisGeNEtTscore 0.2) and CDH23 (DisGeNET score 0.1). We further confirmed the AS events in these genes by visualizing the read coverage via the sashimi tool [31]. Figure 5 demonstrates that the AS events exist in both genes and are consistent between all heart (DCM) samples.

**Figure 5.**
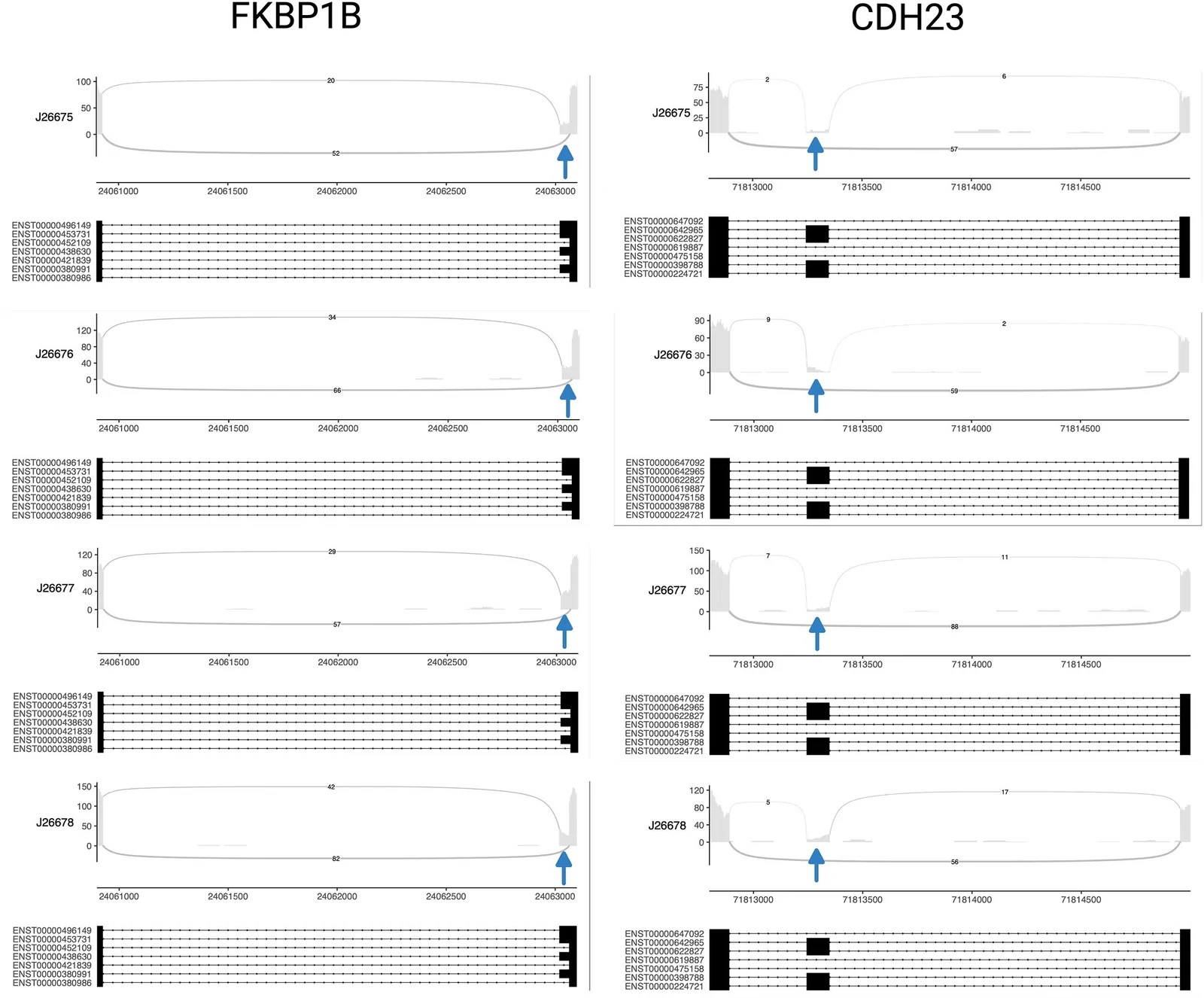
Sashimi plots for low-expressed genes FKBP1B and CDH23 with AS detected only at 200 M reads. The arrows indicate alternative splice site (FKBP1B) and exon skipping (CDH23) events.

Finally, we performed AS-aware functional enrichment analysis using NEASE [30] with KEGG pathways on the exon skipping events detected at 50, 100, 150, and 200 M. We extracted the pathways that could be detected only at 200 M reads (Supplementary Table S7). Among KEGG pathways for the adipose sample before the treatment, we found “fatty acid degradation” (p-adjusted 0.043). For the endotoxin-treated adipose sample, we found the response to viral infections (p-adjusted 0.01). Finally, for the hypothalamus sample, the KEGG pathways list contains terms related to the neural system such as “gap junction” (p-adjusted 0.01), adherens junction (p-adjusted 0.02), and glutamatergic synapse (p-adjusted 0.02).

Altogether, the analysis of genes with AS that can be detected only at 200 M reads reveals that the biologically relevant signals might be weak but still present and worth investigating for a more comprehensive view.

### Comparison of GTEx and TCGA cohorts with the deep-sequenced cohorts

The GTEx [19] and TCGA cohorts comprise a large amount of RNA-seq data from healthy individuals and patients with cancer, respectively. The usual sequencing depth for those cohorts varies between 50 and 150 M reads. As we showed on the SARS-CoV-2 dataset, such sequencing depth might not be enough for a comprehensive AS analysis. We compared the number of genes/junctions with AS we can recover from the GTEx and TCGA RNA-seq data with the amount that could be recovered with the higher sequencing depth estimated from the deep-sequenced cohorts. For the estimation, we used the most simple assumption that the number of events would increase linearly or quadratically.

For the GTEx cohort analysis, we used 102 samples from the hypothalamus with a sequencing depth of 10–236 M reads. We detected AS in these samples and used the number of detections to estimate the number of genes/junctions involved in AS for higher sequencing depth (Fig. 5) using a polynomial fit of degree 1 (blue dashed line) and 2 (orange dashed line). We also approximated these values based on the number of genes/junctions involved in AS detected in samples after downsampling the hypothalamus sample with 236 M reads disregarding the gene expression level (green dashed line). The resulting number of detected genes and junctions has also been used in a polynomial fit of a degree 2.

Figure 6 (top) demonstrates the possible loss in AS detection in the GTEx dataset. As the baseline (black lines), we used the number of genes/junctions that would be detected in a sample with a sequencing depth equal to the mean sequencing depth of the hypothalamus GTEx dataset of 41 M. Analysis of a sample with the mean sequencing depth would result in the possible loss of from up to 7489 genes with AS/114 717 alternative junctions.

**Figure 6.**
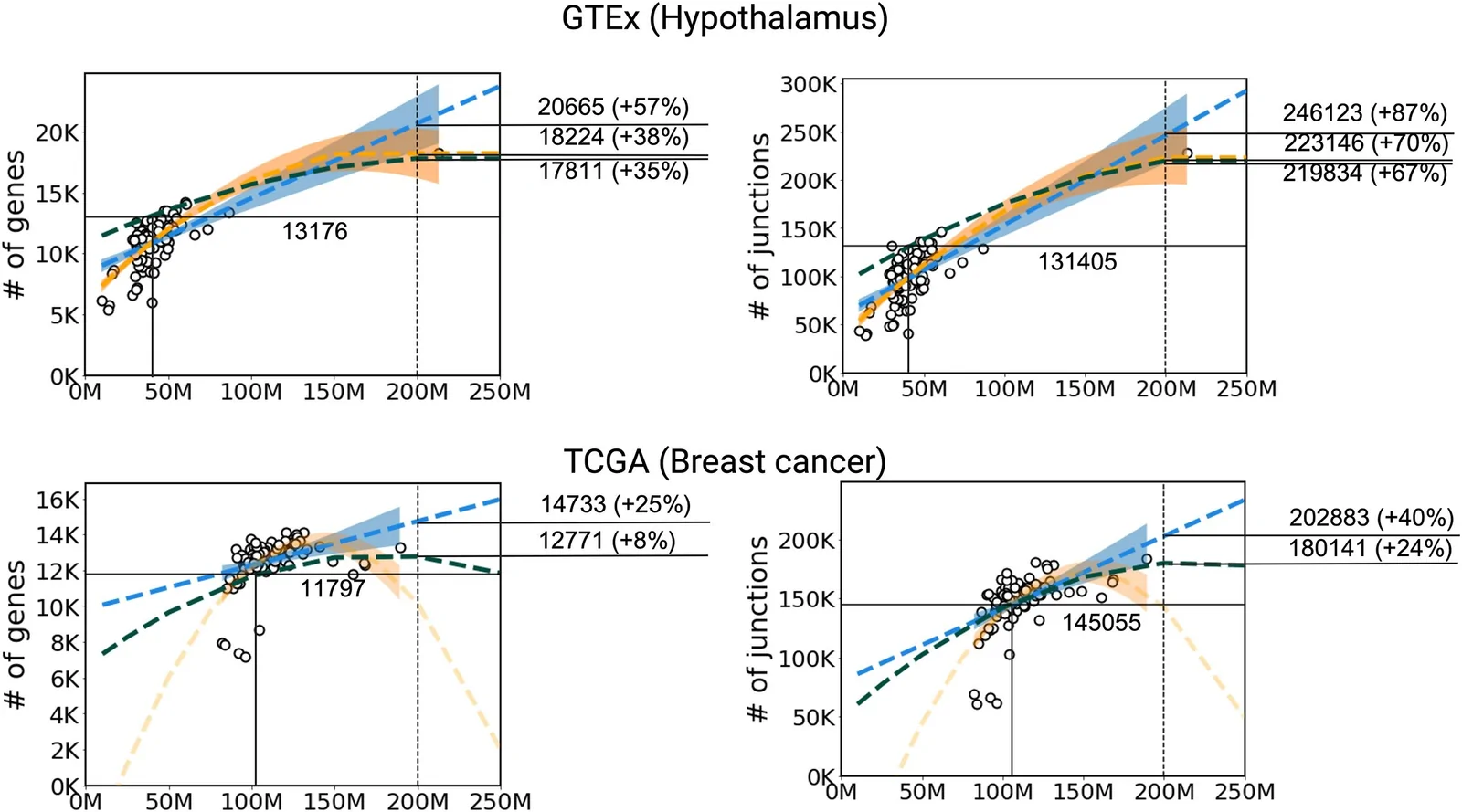
The real (dots) and estimated (dashed lines) number of genes with AS (left) and junctions involved in AS (right) in GTEx hypothalamus and TCGA breast cancer datasets. The number indicates the number of detections at a baseline (black horizontal line), estimated from the downsampling analysis (green dashed line), and estimated from the real datasets using the polynomial fit with the degrees of freedom equal to 1 (blue dashed line) and 2 (orange dashed line). For the percentage, the number of detections in a baseline corresponds to 100%. The shaded area indicates a confidence interval.

For the TCGA breast cancer analysis, we randomly selected 100 samples from the breast cancer cohort and detected genes with AS and alternative junctions. We estimated the number of genes with AS/alternative junctions for higher sequencing depth using a polynomial fit of degree 1 (blue dashed line) and 2 (orange dashed line). Unfortunately, the polynomial fit of degree 2 failed to approximate the number of genes/junctions involved in AS as there were no samples of >200 M reads. Thus, for the TCGA dataset, we performed the downsampling analysis similar to the analysis of deep-sequenced datasets using the sample with the maximum number of reads found in this part of the cohort equal to 189 M. We used the results of the downsampling to approximate the increase in the number of genes/ junctions involved in AS with the increase of sequencing depth by the polynomial that fits a degree of 2 (green dashed line).

Figure 6 (bottom) demonstrates the possible loss in AS detection in the TCGA breast cancer cohort. As the baseline (horizontal black line), we also used the number of genes/junctions that would be detected in a sample with a sequencing depth equal to the mean sequencing depth of 109 M. Analysis of a sample with the mean sequencing depth would result in the possible loss of up to 2935 genes with AS/57 827 alternative junctions.

Summarizing, both of these RNA-seq datasets are undersequenced for comprehensive AS analysis, especially the samples in the GTEx cohort.

### Sequence depth estimator

The linear and quadratic approximation from deep-sequenced cohorts might help to estimate the desired sequencing depth for future studies. We implemented an online sequence depth estimator to support the choice. A user can choose the dataset for the approximation, the confidence level (in how many subsamples the event should be detected), and the percentage of events they would like to detect. The estimator is available at https://sequencing-depth4splicing.com/.

### The cost of AS detection in the deep-sequenced cohorts

Experiments with >200 M reads might be economically impractical. We used the prices published on the CCGA Kiel website and estimated/calculated that the cost for each additional detection (a gene or a junction with AS) grows notably after 200–250 M reads (Fig. 7). Together with the results from deep-sequenced cohorts, this result supports the threshold of 200 M reads as an optimal trade-off between costs and benefits for AS analysis.

**Figure 7.**
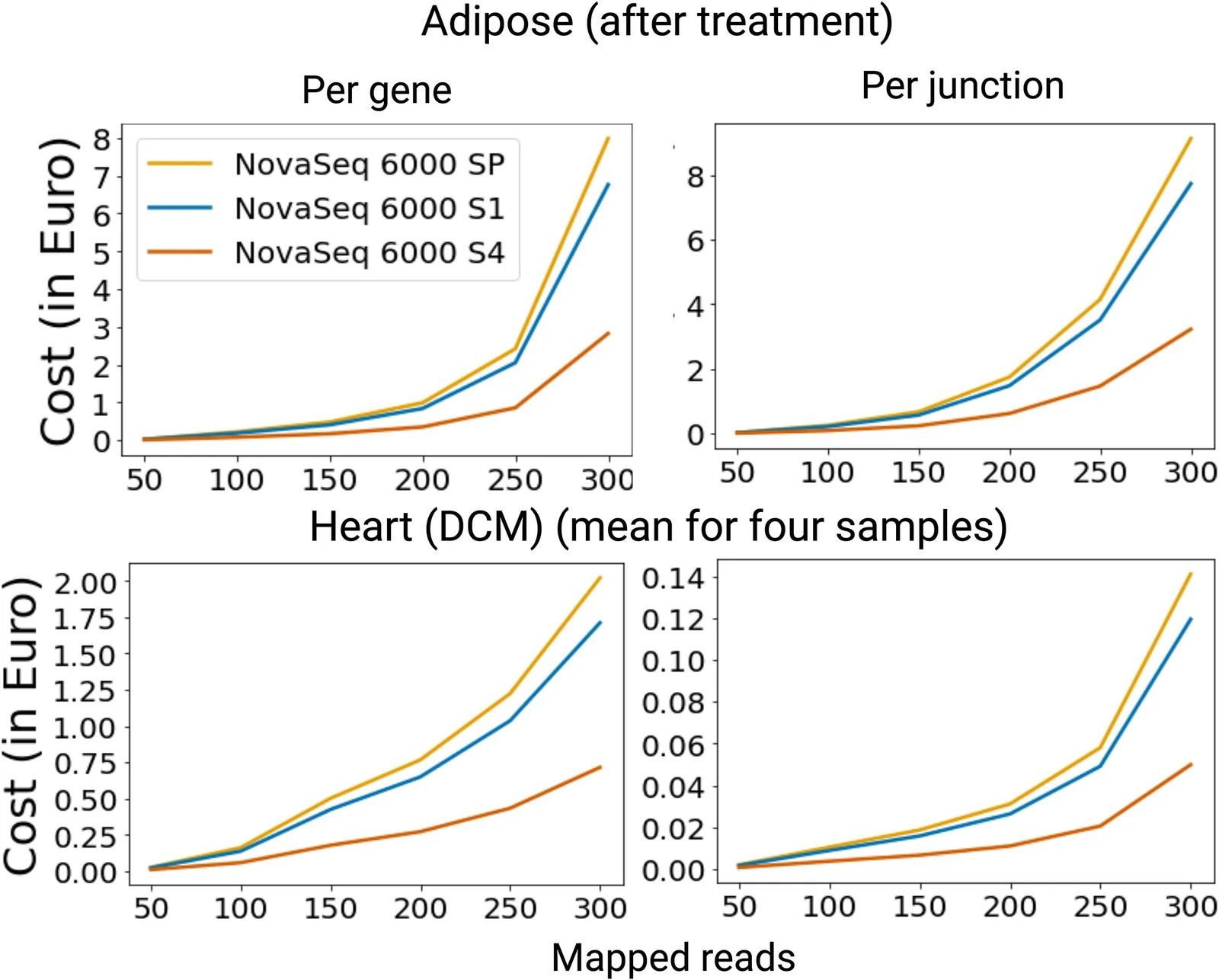
The cost for each additional gene with AS and junction detected while increasing the number of mapped reads.

## Discussion

The appropriate choice of sequencing depth is crucial for successful RNA-seq analysis, especially when studying AS. Our results demonstrate that the threshold depends on the purpose of the study: to detect all AS events, the threshold should be set to 150–200 M reads for lowly expressed genes and 100–150 M for highly expressed genes. Thus, we observed the increase of new AS detections to be <1% after these thresholds. Other gene characteristics (such as gene region) influence the detection of AS, but their impact is much smaller than that of gene expression levels.

Several aspects need to be considered when interpreting this threshold. The sequencing depth of a sample is typically higher than the number of mapped reads; however, in high-quality experiments, those numbers should be close to each other. We defined the threshold based on short-read paired-end mRNA RNA-seq data from human data, and we should note that experimental design might impact the results [13]. First, the choice of sequencing technology and library preparation affects this threshold. For example, single-end short sequencing data, which is less robust for gene and isoform expression analysis [7], likely has different thresholds. Read length and the level of RNA degradation might also impact the threshold for AS detection: shorter length decreases the fraction of uniquely mapped reads. Consistent with this, Ladwig *et al*. showed that longer reads and higher read depth increase the information available for MAJIQ-based splicing analysis, including the detection of local splicing variations and regulated events. Thus, the sequencing-depth thresholds proposed here should be interpreted in the context of the short-read paired-end datasets analyzed in this study, which had read lengths of 50–150 bp. Concerning RNA degradation, in our study, we did not address its effect on AS detection, as the analyzed datasets present high-quality data [the RIN (RNA integrity number) value 8–10], and the datasets with the RIN value <7 is generally not recommended to use for the transcriptome analysis.

Similarly, we expect this threshold to differ for long-read sequencing techniques that can detect the full-length and novel transcripts. However, despite the rapid development of long-read sequencing technology, like the increase in base calling and transcript identification accuracy [34], this technology is currently more expensive and requires the extraction of a larger amount of undamaged nucleotide material that might be challenging. Thus, short-read sequencing technologies are currently more widely used for quantitative AS analysis and present the best option in terms of cost-effectiveness and requirements for the material preparation.

Another limitation of this study is the selection of the organism: all found deep-sequenced RNA-seq datasets have been derived from the human samples. For other organisms, the threshold might differ because of the difference in splicing patterns, e.g. in humans, exon skipping was shown to be the prevalent AS event but in plants, intron retention prevails [35]. However, the threshold for the sequencing depth defined in our study is robust regardless of disease or tissue type.

Finally, our results may be affected by the computational workflow selected. While the mapping tool should not impact the results, choosing an AS detection tool might impact the threshold as the number of detected AS events differs notably between tools [6]. However, a previous study on human adipose tissue with a different AS detection tool—MATS— demonstrated similar results [8].

Before choosing the number of reads for a particular experiment, one more important question is how relevant AS events detected only at deeper sequencing could be to the phenotype/disease of interest. Deveson *et al*. [36] demonstrated that with increasing sequencing depth noncoding introns and isoforms also kept increasing. However, the event level resolution we use in the study cannot consider if the resulting isoform will be coding or not. On the gene level, we do not see any prevalence of AS events in the nonprotein-coding genes (data not shown). Our study demonstrated that the higher sequencing depth results in more AS events in genes with low and high (but not very high) expression levels. Each of these events brings only a subtle change. Still, if they accumulate in one metabolic pathway or protein complex, the additive effect might lead to the phenotype/disease onset. For example, we demonstrated for the hypothalamus and adipose datasets, that the changes detected at higher sequencing depth accumulate in phenotype-specific pathways. The low-expressed genes that carry functionally relevant AS events could be further investigated using the targeted approaches, such as hybridization capture kits (e.g. myBaits from Arbor Biosciences). Our results suggest that the threshold of 150–200 M reads for lowly and 100–150 M highly expressed genes present a trade-off between the number of detections to reveal the role of AS in the phenotype/disease of interest and cost-effectiveness. Thus, the typical threshold for sequencing depth chosen in contemporary human RNA-seq studies e.g. TCGA and GTEx is too low for a complete assessment of the AS landscape, and future projects may consider sequencing deeper to better support AS analysis.

## Supplementary Material

gkag653_Supplemental_File

## Data Availability

The preprocessed data underlying this article are available in Zenodo at https://doi.org/10.5281/zenodo.11655945. The GTEx data underlying this article were obtained from dbGaP accession number phs000424.GTEx.v8.p2 (https://dbgap.ncbi.nlm.nih.gov/beta/study/phs000424.v11.p2) on 21/02/2020. The TCGA data used here are, in part, based upon data generated by the TCGA Research Network: https://www.cancer.gov/tcga. The SARS-CoV-2 and human adipose RNA-seq data are available in Gene Expression Omnibus (GEO) [18] at https://www.ncbi.nlm.nih.gov/Traces/study/?acc=PRJNA787951 and https://www.ncbi.nlm.nih.gov/Traces/study/?acc=PRJNA198737 and can be accessed with GSE190680, GSE46323. The Heart (DCM) RNA-seq data will be shared on reasonable request to the corresponding author via voelker@uni-greifswald.de. All Python scripts used to generate the figures are available at https://github.com/OlgaVT/RNA-seq_Depth_for_AS and https://doi.org/10.5281/zenodo.11655945.

## References

[1] Sims D, Sudbery I, Ilott NE et al. Sequencing depth and coverage: key considerations in genomic analyses. Nat Rev Genet. 2014;15:121–32. 10.1038/nrg3642 24434847

[2] Trincado JL, Entizne JC, Hysenaj G et al. SUPPA2: fast, accurate, and uncertainty-aware differential splicing analysis across multiple conditions. Genome Biol. 2018;19:40. 10.1186/s13059-018-1417-1 PMC5866513 29571299

[3] Trapnell C, Hendrickson DG, Sauvageau M et al. Differential analysis of gene regulation at transcript resolution with RNA-seq. Nat Biotechnol. 2013;31:46–53. 10.1038/nbt.2450 PMC3869392 23222703

[4] Wu W, Zong J, Wei N et al. CASH: a constructing comprehensive splice site method for detecting AS events. Brief Bioinform. 2018;19:905–17. 10.1093/bib/bbx034 28387786

[5] Manz Q, Tsoy O, Fenn A et al. ASimulatoR: splice-aware RNA-seq data simulation. Bioinformatics. 2021;37:3008–10. 10.1093/bioinformatics/btab142 33647976

[6] Fenn A, Tsoy O, Faro T et al. AS analysis benchmark with DICAST. NAR Genom Bioinform. 2023;5:lqad044. 10.1093/nargab/lqad044 PMC10227362 37260511

[7] Tarazona S, García-Alcalde F, Dopazo J et al. Differential expression in RNA-seq: a matter of depth. Genome Res. 2011;21:2213–23. 10.1101/gr.124321.111 PMC3227109 21903743

[8] Liu Y, Ferguson JF, Xue C et al. Evaluating the impact of sequencing depth on transcriptome profiling in human adipose. PLoS One. 2013;8:e66883. 10.1371/journal.pone.0066883 PMC3691247 23826166

[9] Bass AJ, Robinson DG, Storey JD. Determining sufficient sequencing depth in RNA-seq differential expression studies. bioRxiv, 10.1101/635623, 13 May 2019, preprint: not peer reviewed.

[10] SEQC/MAQC-III Consortium . A comprehensive assessment of RNA-seq accuracy, reproducibility and information content by the Sequencing Quality Control Consortium. Nat Biotechnol. 2014;32:903–14. 10.1038/nbt.2957 PMC4321899 25150838

[11] Jiang L, Schlesinger F, Davis CA et al. Synthetic spike-in standards for RNA-seq experiments. Genome Res. 2011;21:1543–51. 10.1101/gr.121095.111 PMC3166838 21816910

[12] Hardwick SA, Chen WY, Wong T et al. Spliced synthetic genes as internal controls in RNA sequencing experiments. Nat Methods. 2016;13:792–8. 10.1038/nmeth.3958 27502218

[13] Ladwig A, Klostermann M, Zarnack K. The impact of read depth and read length on RNA-seq splicing analysis. RNA. 2026;32:993–1004. 10.1261/rna.080915.125 PMC13271005 42020155

[14] Lee HK, Knabl L, Knabl L et al. Immune transcriptome analysis of COVID-19 patients infected with SARS-CoV-2 variants carrying the E484K escape mutation identifies a distinct gene module. Sci Rep. 2022;12:2784. 10.1038/s41598-022-06752-0 PMC8857234 35181735

[15] Lee S-G, Furth PA, Hennighausen L et al. Variant- and vaccination-specific AS profiles in SARS-CoV-2 infections. iScience. 2024;27:109177. 10.1016/j.isci.2024.109177 PMC10897911 38414855

[16] Lee HK, Knabl L, Walter M et al. Prior vaccination exceeds prior infection in eliciting innate and humoral immune responses in Omicron infected outpatients. Front Immunol. 2022;13:916686. 10.3389/fimmu.2022.916686 PMC9240221 35784346

[17] Hoffmann M, Willruth L-L, Dietrich A et al. Blood transcriptomics analysis offers insights into variant-specific immune response to SARS-CoV-2. Sci Rep. 2024;14:2808. 10.1038/s41598-024-53117-w PMC10837437 38307916

[18] Edgar R, Domrachev M, Lash AE. Gene expression omnibus: NCBI gene expression and hybridization array data repository. Nucleic Acids Res. 2002;30:207–10. 10.1093/nar/30.1.207 PMC99122 11752295

[19] GTEx Consortium The genotype-tissue expression (GTEx) project. Nat Genet. 2013;45:580–5. 10.1038/ng.2653 PMC4010069 23715323

[20] Dobin A, Davis CA, Schlesinger F et al. STAR: ultrafast universal RNA-seq aligner. Bioinformatics. 2013;29:15–21. 10.1093/bioinformatics/bts635 PMC3530905 23104886

[21] Baruzzo G, Hayer KE, Kim EJ et al. Simulation-based comprehensive benchmarking of RNA-seq aligners. Nat Methods. 2017;14:135–9. 10.1038/nmeth.4106 PMC5792058 27941783

[22] Liao Y, Smyth GK, Shi W. featureCounts: an efficient general purpose program for assigning sequence reads to genomic features. Bioinformatics. 2014;30:923–30. 10.1093/bioinformatics/btt656 24227677

[23] Li H, Handsaker B, Wysoker A. et al. The sequence alignment/map format and SAMtools. Bioinformatics. 2009;25:2078–9. 10.1093/bioinformatics/btp352 PMC2723002 19505943

[24] Cotto KC, Feng Y-Y, Ramu A et al. Integrated analysis of genomic and transcriptomic data for the discovery of splice-associated variants in cancer. Nat Commun. 2023;14:1589. 10.1038/s41467-023-37266-6 PMC10033906 36949070

[25] Vaquero-Garcia J, Barrera A, Gazzara MR et al. A new view of transcriptome complexity and regulation through the lens of local splicing variations. eLife. 2016;5:e11752. 10.7554/eLife.11752 PMC4801060 26829591

[26] Ahn J, Wu H, Lee K. Integrative analysis revealing human heart-specific genes and consolidating heart-related phenotypes. Front Genet. 2020;11:500277. 10.3389/fgene.2020.00777 PMC7438927 32903789

[27] Pollard KS, Salama SR, King B et al. Forces shaping the fastest evolving regions in the human genome. PLoS Genet. 2006;2:e168. 10.1371/journal.pgen.0020168 PMC1599772 17040131

[28] Nellore A, Jaffe AE, Fortin JP et al. Human splicing diversity and the extent of unannotated splice junctions across human RNA-seq samples on the sequence read archive. Genome Biol. 2016;17:266. 10.1186/s13059-016-1118-6 PMC5203714 28038678

[29] Kolberg L, Raudvere U, Kuzmin I et al. g:Profiler-interoperable web service for functional enrichment analysis and gene identifier mapping (2023 update). Nucleic Acids Res. 2023;51:W207–12. 10.1093/nar/gkad347 PMC10320099 37144459

[30] Louadi Z, Elkjaer ML, Klug M et al. Functional enrichment of AS events with NEASE reveals insights into tissue identity and diseases. Genome Biol. 2021;22:327. 10.1186/s13059-021-02538-1 PMC8638120 34857024

[31] Garrido-Martín D, Palumbo E, Guigó R et al. ggsashimi: sashimi plot revised for browser- and annotation-independent splicing visualization. PLoS Comput Biol. 2018;14:e1006360. 10.1371/journal.pcbi.1006360 PMC6114895 30118475

[32] Hart SN, Therneau TM, Zhang Y et al. Calculating sample size estimates for RNA sequencing data. J Comput Biol. 2013;20:970–8. 10.1089/cmb.2012.0283 PMC3842884 23961961

[33] Piñero J, Ramírez-Anguita JM, Saüch-Pitarch J et al. The DisGeNET knowledge platform for disease genomics: 2019 update. Nucleic Acids Res. 2020;48:D845–55. 10.1093/nar/gkz1021 PMC7145631 31680165

[34] Pardo-Palacios FJ, Wang D, Reese F et al. Systematic assessment of long-read RNA-seq methods for transcript identification and quantification. bioRxiv, 10.1101/2023.07.25.550582, 27 July 2023, preprint: not peer reviewed. PMC11543605 38849569

[35] Ner-Gaon H, Halachmi R, Savaldi-Goldstein S et al. Intron retention is a major phenomenon in AS in *Arabidopsis*. Plant J. 2004;39:877–85. 10.1111/j.1365-313X.2004.02172.x 15341630

[36] Deveson IW, Brunck ME, Blackburn J et al. Universal alternative splicing of noncoding exons. Cell Syst. 2018;6:245–55.e5. 10.1016/j.cels.2017.12.005 29396323

