# Supplementary material for: RNA sequencing depth guidelines for the study of alternative splicing
Source: Nucleic Acids Res. 2026 Nov 11;54(21):gkag653. doi: 10.1093/nar/gkag653 (PMC13752507; doi:10.1093/nar/gkag653)
Supplement: Supplementary file 1 [file gkag653_supplemental_file.pdf]

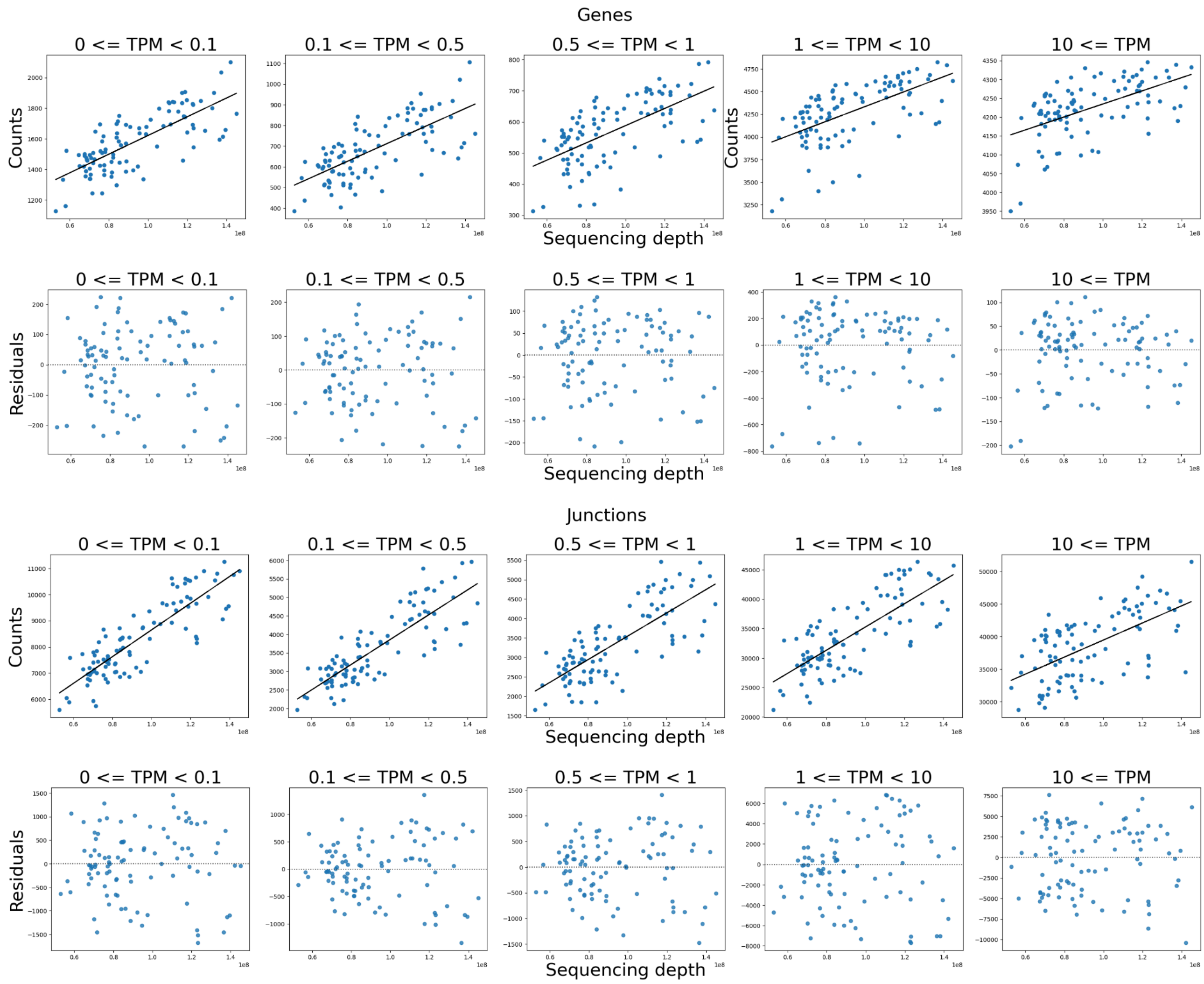

Supplementary Figure 1. Linear regression and residual plots for the dependence between number of genes with alternative splicing events (top) and junctions involved in alternative splicing events (bottom) and sequencing depth grouped by expression level.

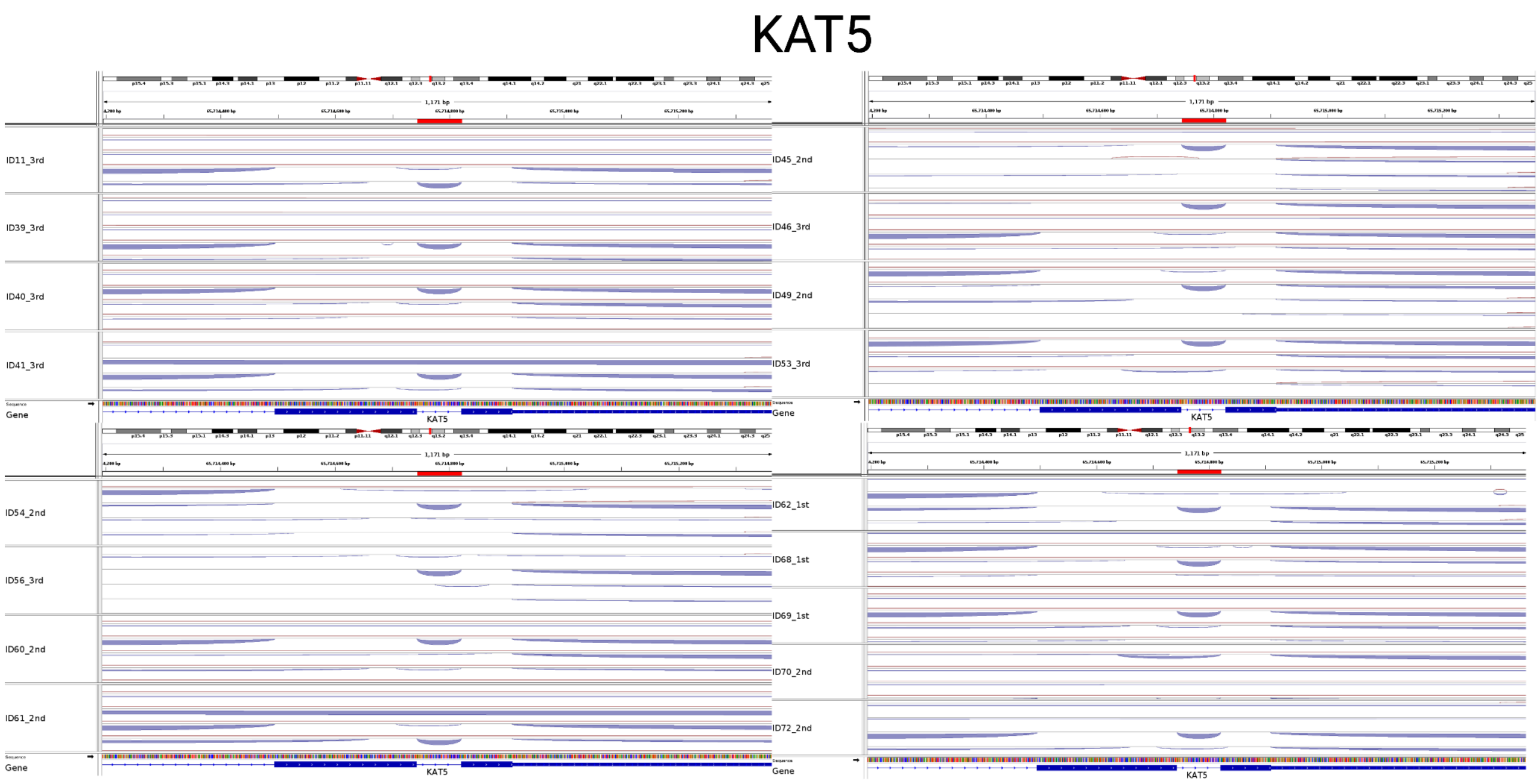

KIF21A

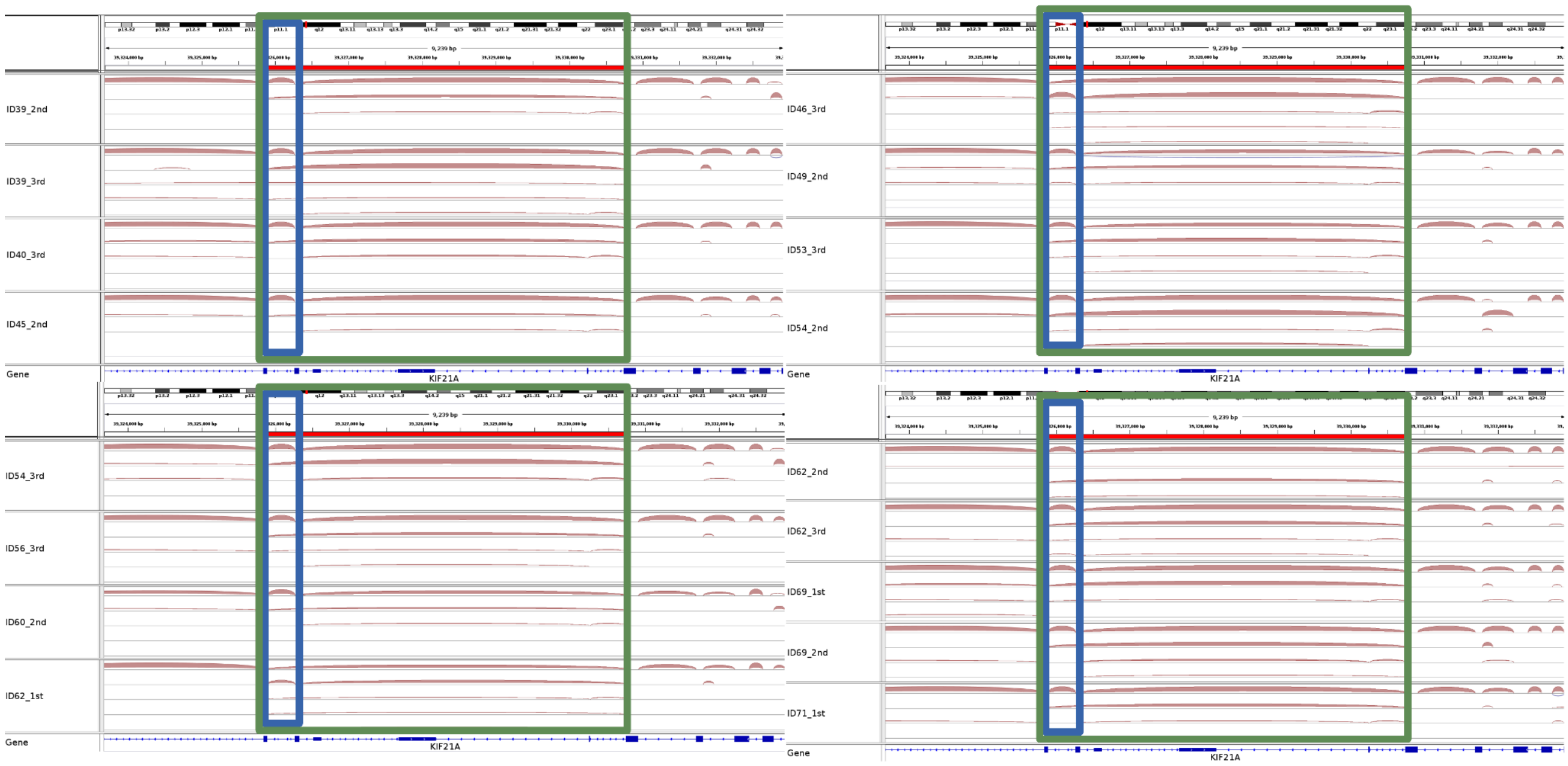

NR6A1

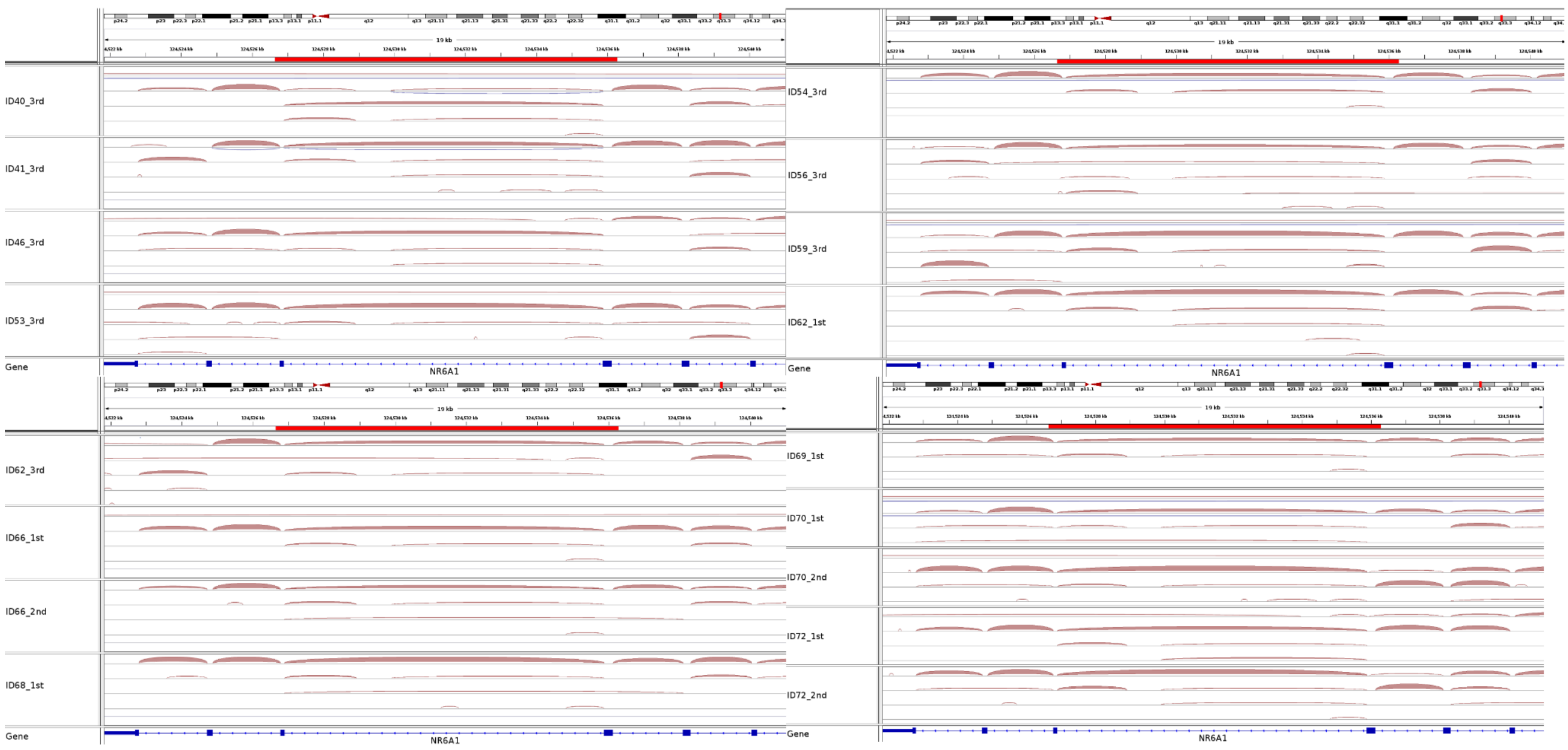

PRKACB

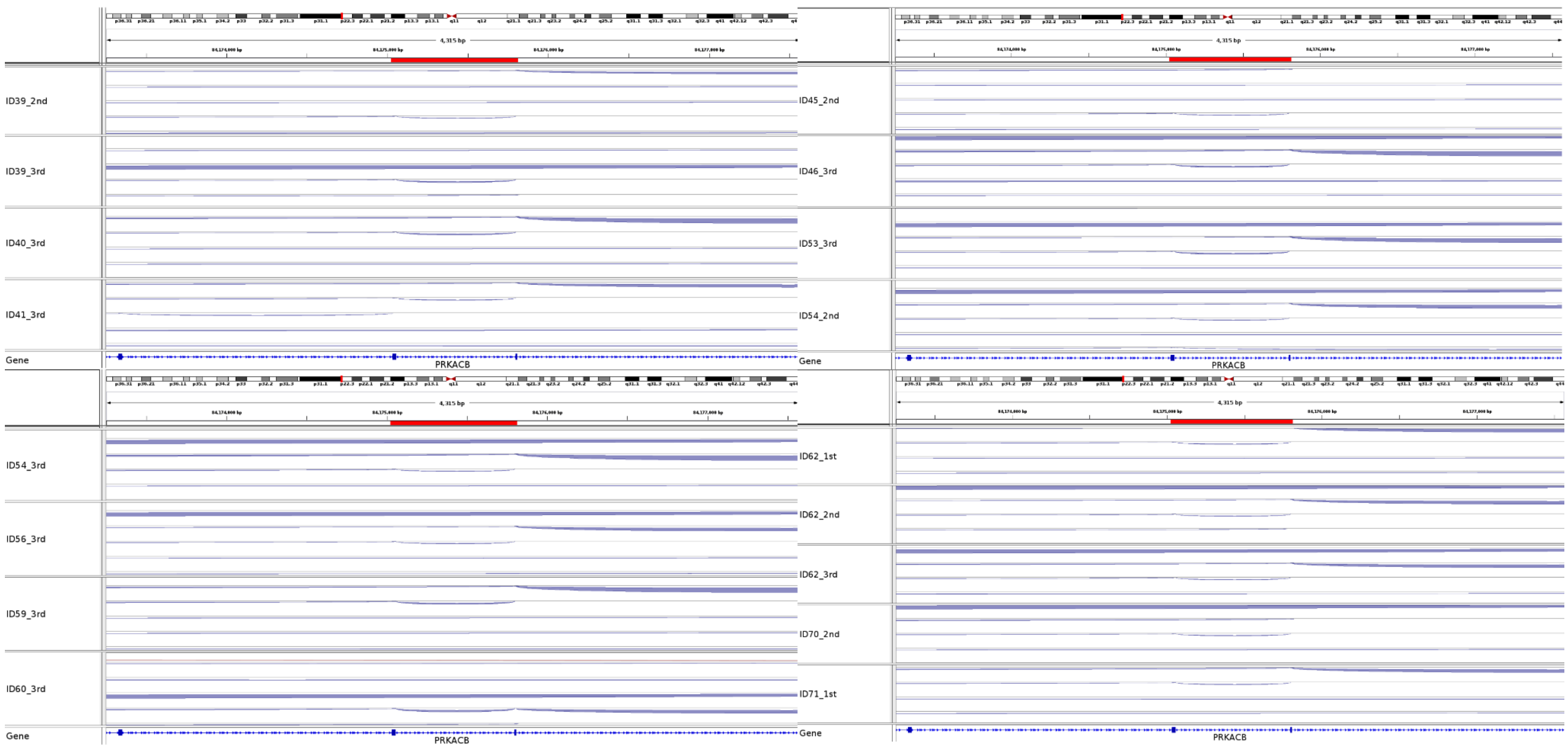

TP53BP2

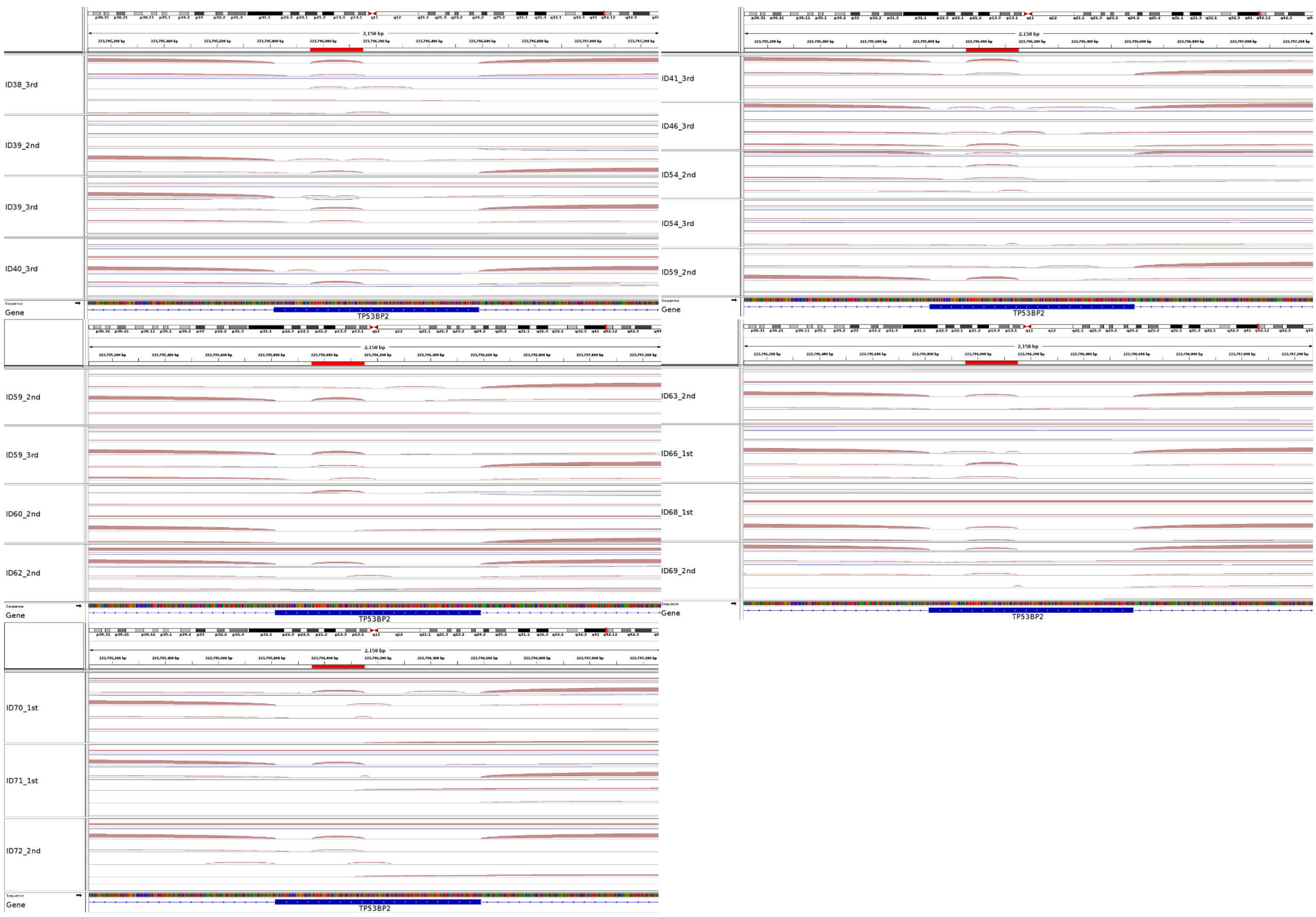

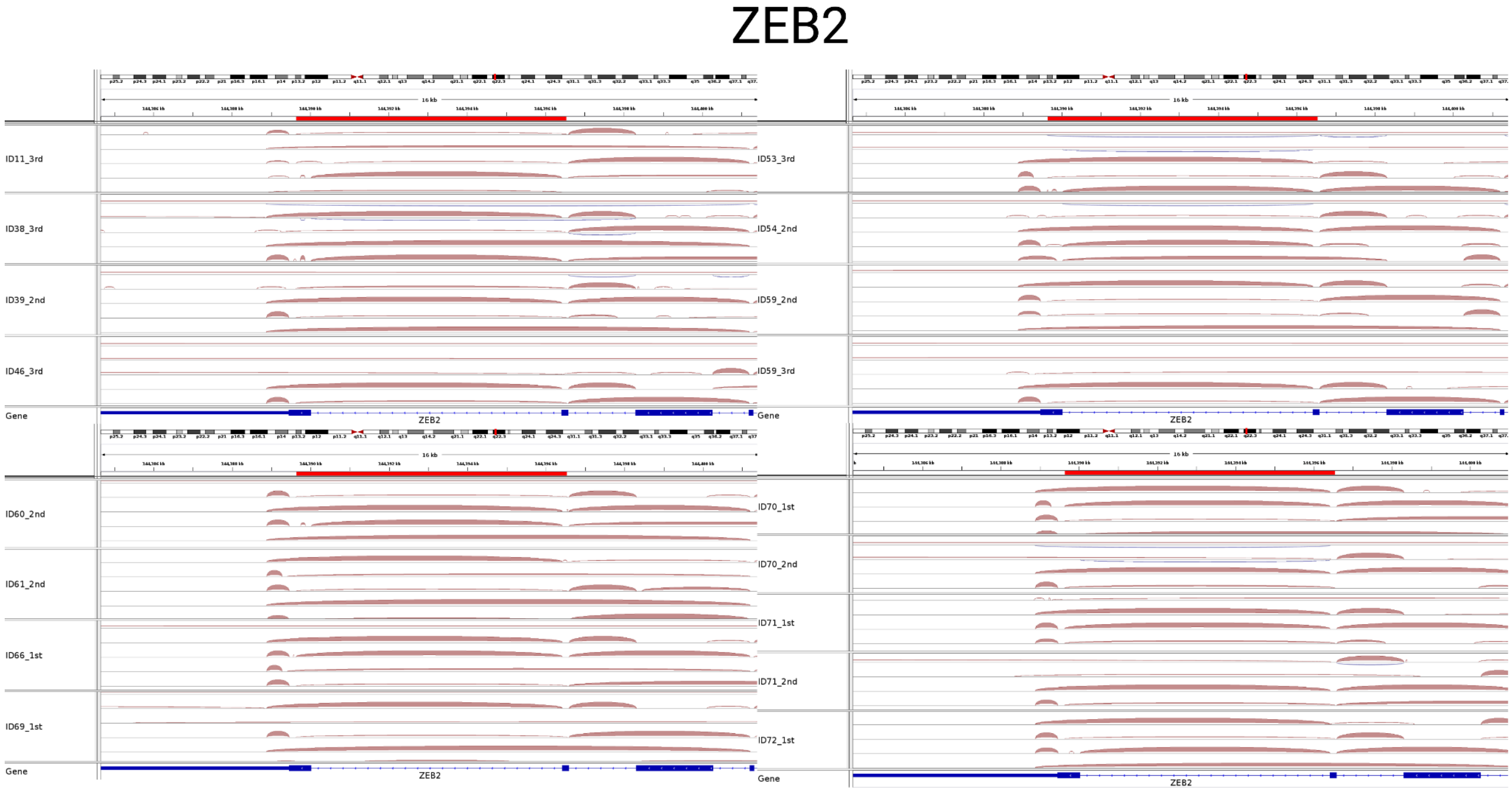

Supplementary Figure S2. Read coverage of junctions detected to be involved in AS only in samples with a higher sequencing depth.

The red line marks the coordinates of the alternative junction and the plots below represent the coverage that supports these junctions from the alignment bam files per each sample.

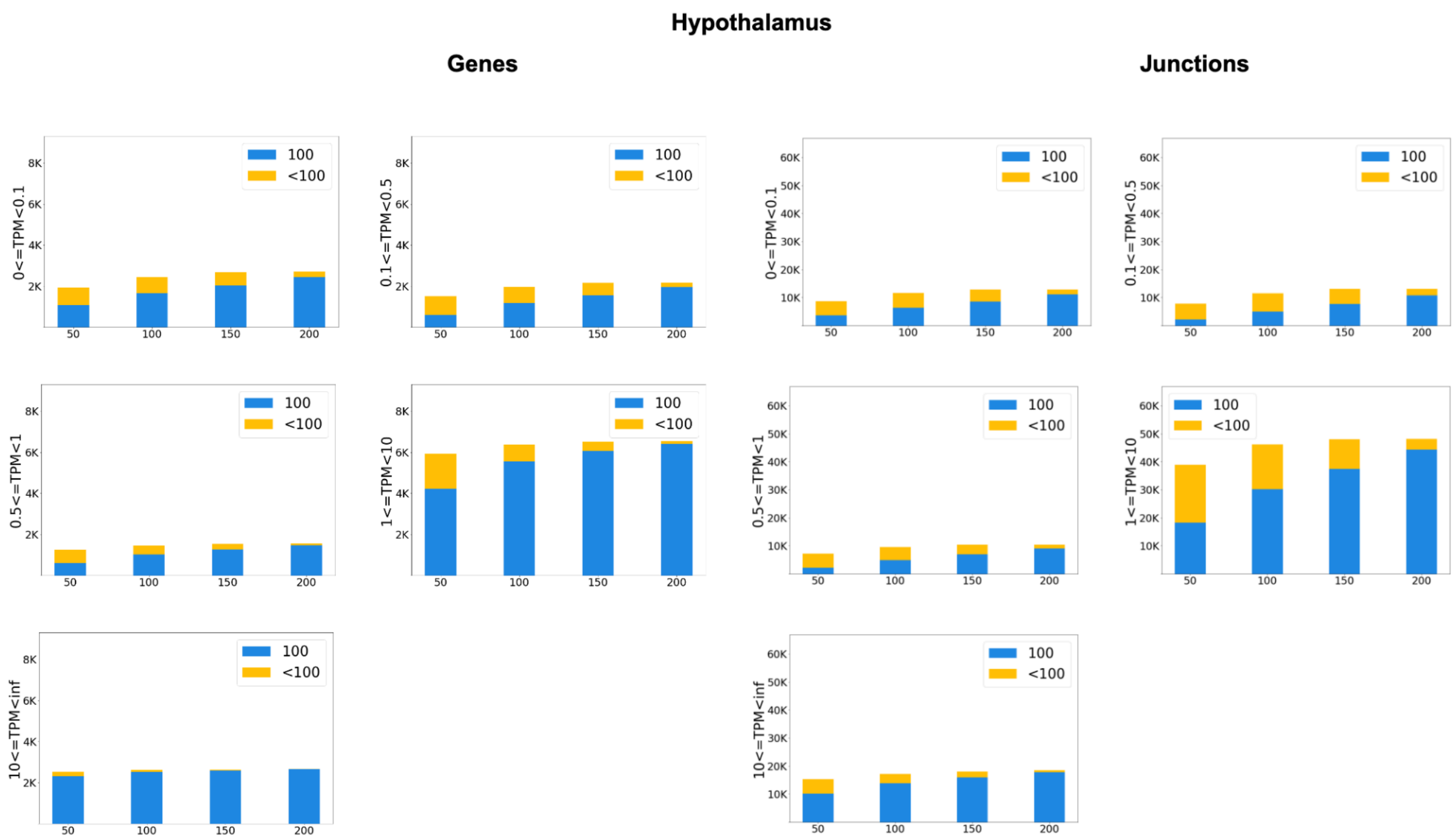

Supplementary Figure S3. The number of genes and junctions involved in AS detected in 100 (blue), and less than 100 (yellow) out of 100 subsamples for Hypothalamus. Each stacked bar depicts one sample.

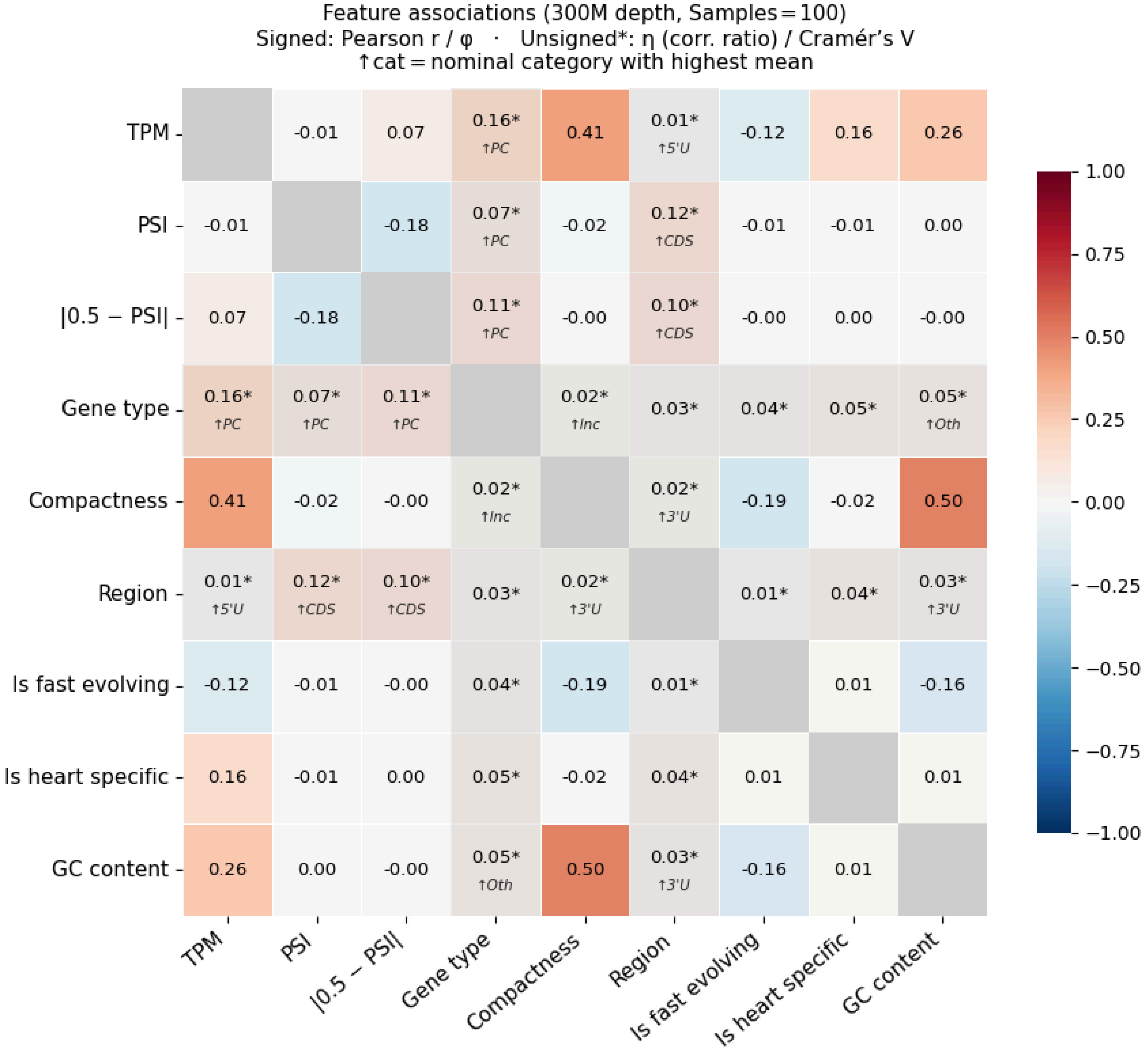

Supplementary Figure S4. Pairwise feature associations. Mixed-association matrix of the nine features of junctions and junction-containing genes. Correlation method depends on the variable pair: signed Pearson r (or point-biserial r) is used for continuous–continuous, continuous–boolean, and boolean–boolean pairs (colour scale –1 to +1); the unsigned correlation ratio η is used for continuous–nominal pairs; Cramér's V is used for nominal–nominal and nominal–boolean pairs. Unsigned values are indicated with an asterisk (*) and a grey cell tint. For each continuous–nominal pair, the nominal category with the highest group mean is annotated (↑cat; abbreviations: PC = Protein-coding, lnc = lncRNA, Oth = Other, 3'U = 3′ UTR, 5'U = 5′ UTR).

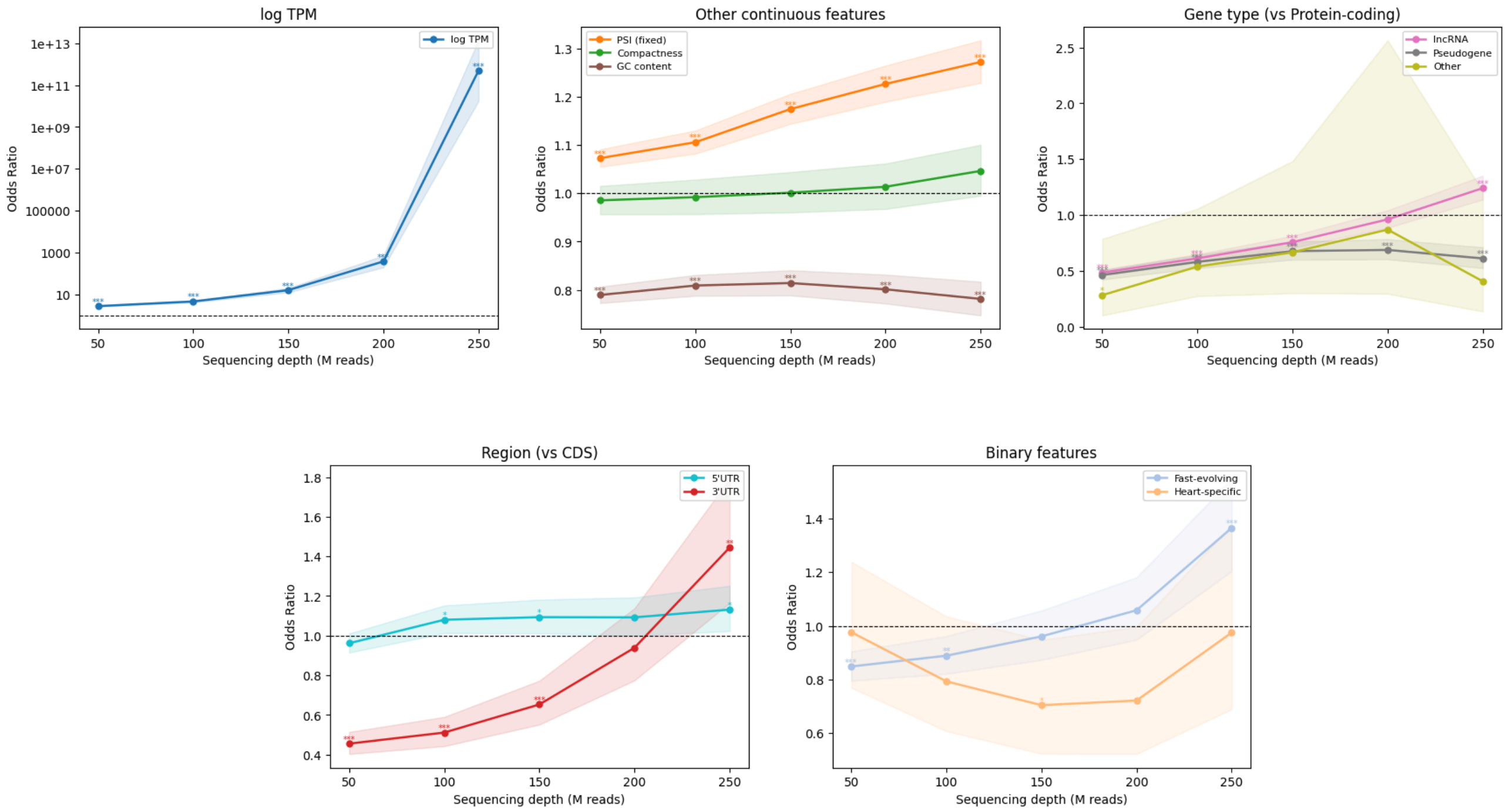

Supplementary Figure S5. GEE-estimated odds ratios for splice-junction detectability across sequencing depths. Odds ratios (OR) and 95% confidence intervals from generalised estimating equations (GEE) modelling the binary outcome of splice-junction detection (detected in ≥1 sample vs. undetected). The odds of detection are defined as P(detected) / P(not detected), and the OR is the multiplicative change in those odds associated with a one-unit increase in the predictor. Because continuous predictors were z-scored prior to modelling, one unit equals one standard deviation, so each OR reflects the effect of moving one SD above the mean. Models were fitted independently at five sequencing depths (50–250 M read pairs; 300 M used as the reference calibration depth but excluded from the plot for clarity). Junctions were restricted to those with PSI 5–95% in at least one patient at 300 M (n = 117,505 junctions; four DCM patients). Continuous predictors (log TPM, PSI, Compactness, GC content) were z-scored using a common scale derived from the full dataset; binary predictors (fast-evolving, heart-specific) were included as-is; gene type was dummy-coded relative to Protein-coding; genomic region (5′UTR, 3′UTR, or blank) was dummy-coded relative to CDS. ORs for log TPM are shown on a log scale; all other panels use a linear scale. Shaded bands indicate 95% CIs. Significance annotations reflect Benjamini–Hochberg-corrected FDR < 0.05/0.01/0.001 within each depth.

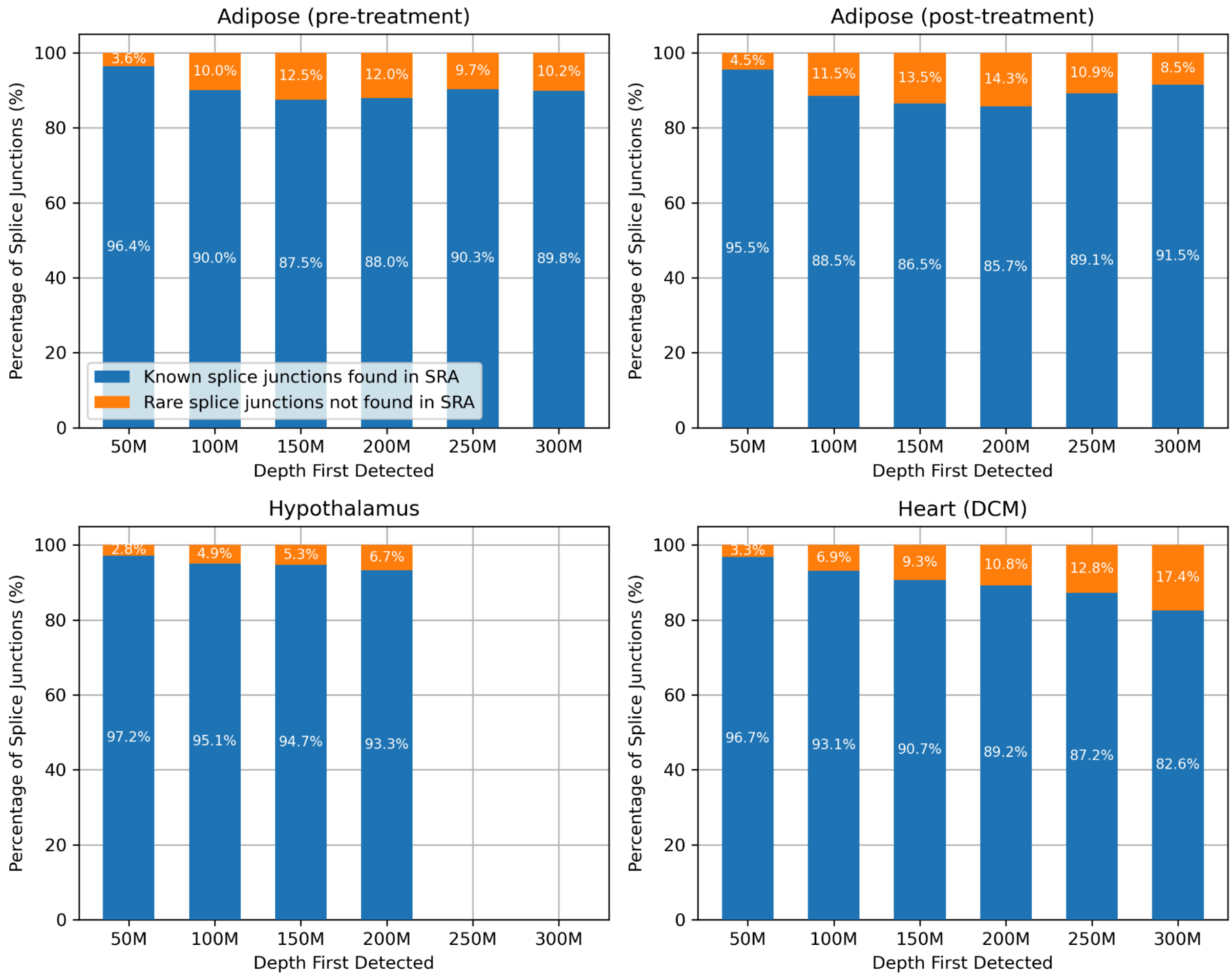

Supplementary Figure S6. The proportion of junctions involved in AS that are detected at a certain RNA-Seq depth for the first time and (blue) found in SRA samples and (orange) not found.

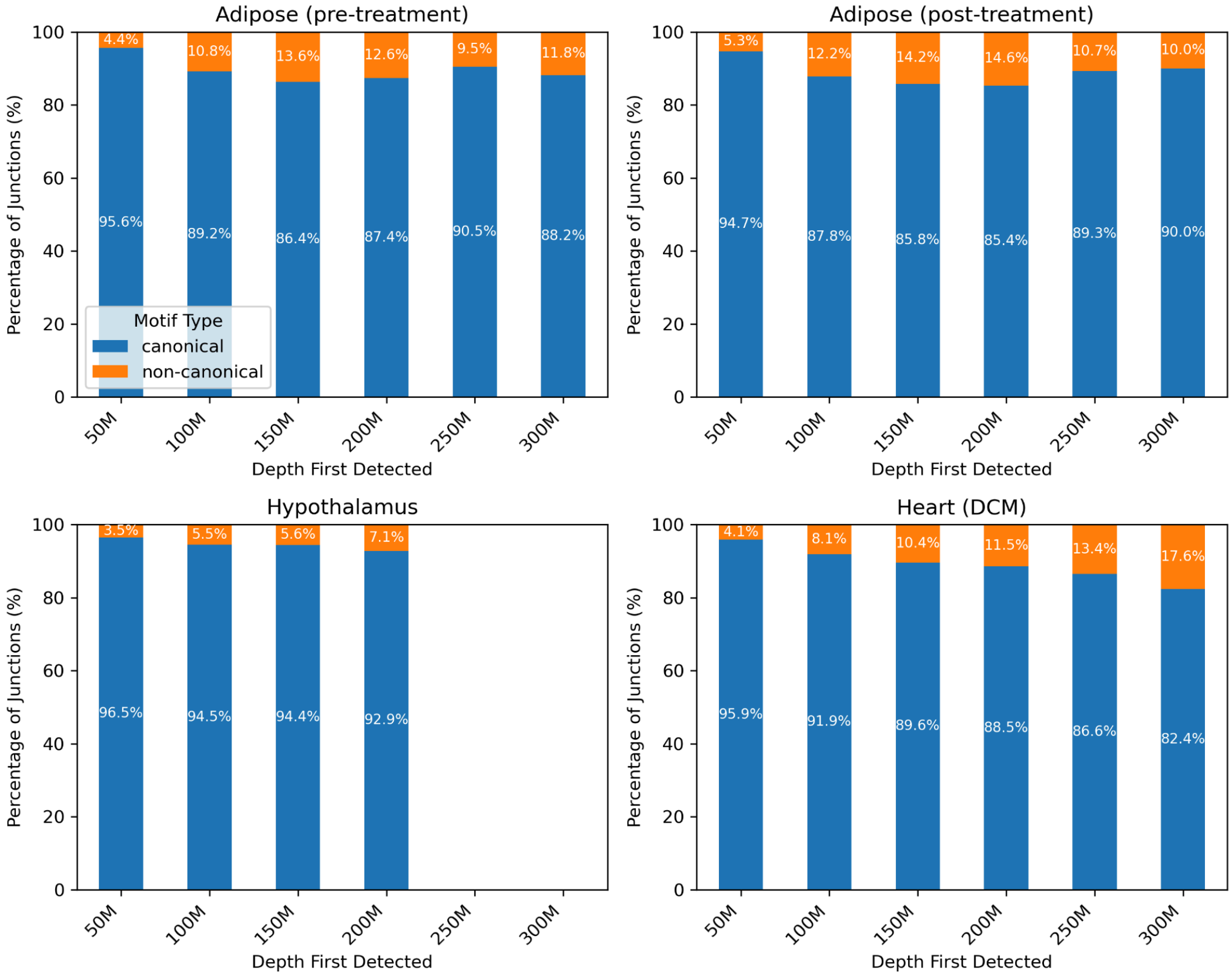

Supplementary Figure S7. The number of junctions involved in AS that are detected at a certain RNA-Seq depth for the first time with (blue) canonical or (orange) non-canonical splice sites.

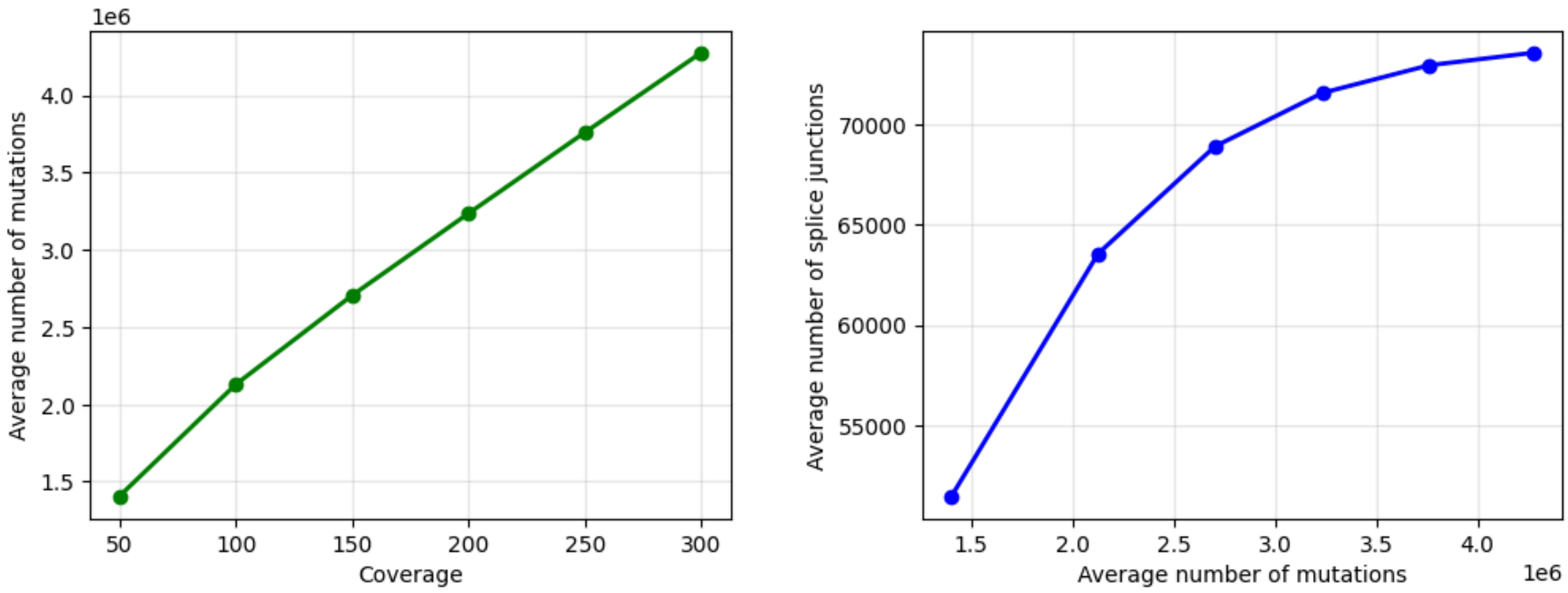

Supplementary Figure S8. Sequencing coverage, mutation burden, and splice-junction discovery. **Left:** Average transcriptome-captured somatic mutations (called with rnaseqmut) vs. RNA-seq coverage, showing a linear increase with depth. **Right:** Average detected splice junctions vs. mutation burden, rising then flattening—indicating diminishing returns beyond coverage.

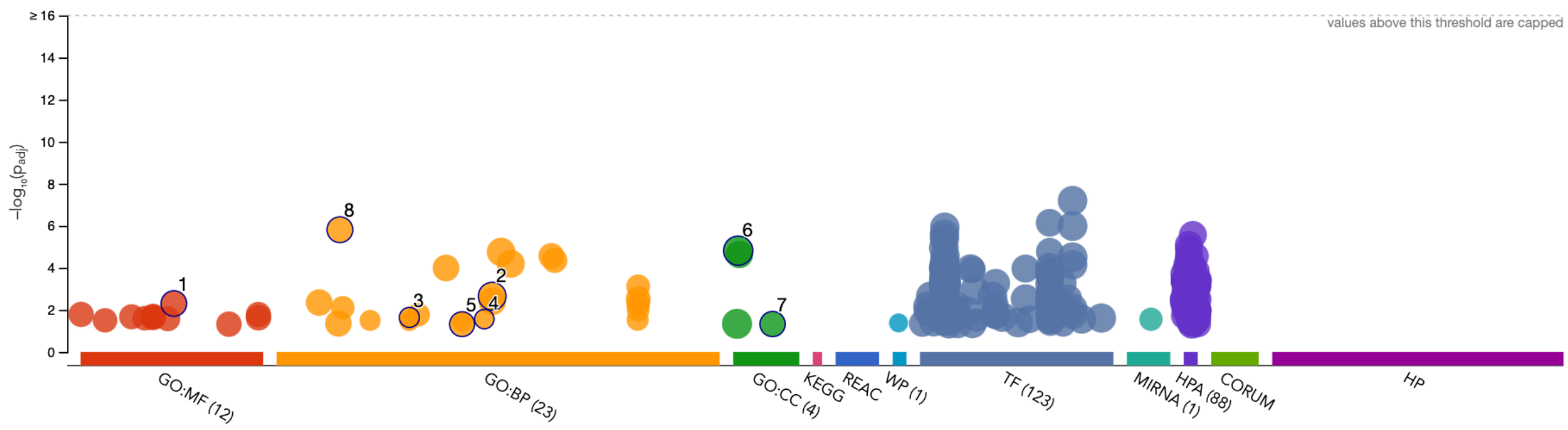

ID	Source	Term ID	Term Name	p_{adj} (query_1)
8	GO:BP	GO:0010033	response to organic substance	1.532×10^{-6}
6	GO:CC	GO:0005737	cytoplasm	1.517×10^{-5}
2	GO:BP	GO:0048518	positive regulation of biological process	2.210×10^{-3}
1	GO:MF	GO:0043168	anion binding	4.950×10^{-3}
3	GO:BP	GO:0032869	cellular response to insulin stimulus	2.269×10^{-2}
4	GO:BP	GO:0046718	symbiont entry into host cell	2.714×10^{-2}
7	GO:CC	GO:0070062	extracellular exosome	4.674×10^{-2}
5	GO:BP	GO:0044419	biological process involved in interspecies inte...	4.748×10^{-2}

Supplementary Figure S9. The results of the gProfiler for the genes with high expression (TPM>1) with AS detected only at 200M reads from the adipose sample after endotoxin treatment.

Supplementary Table S1. Alternative splicing tools that simulated RNA-Seq data at various sequencing depths for the evaluation of the tool performance

Tool name	Maximum sequencing depth of the simulated RNA-Seq data	Reference
ASpedia-FI	52M	(1)
AS-Quant	50M	(2)
ASTool	60M	(3)
iREAD	30M	(4)
splAdder	20 M for 1000 genes	(5)
McSplicer	75M	(6)
FreePSI	50M	(7)
SUPPA2	120M	(8)
NURD	10M	(9)
Cufflinks	100M	(10)
CASH	100M	(11)
EventPointer3.0	125M	(12)
ASimulatoR	200M	(13)

DICAST	200M	(14)

Supplementary Table S2. Number of genes and junctions in each expression category

	SARS-Cov2	Hypothalamus	Adipose (pre-treatment)	Adipose (post-treatment)	Heart (DCM)
	**Genes**				
TPM < 0.1	6149 ± 647	10303	11863	12010	13436 ± 412
0.5 > TPM ≥0.1	4213 ± 158	6179	5394	5249	4889 ± 98
1 > TPM ≥ 0.5	2006 ± 63	2825	2380	2204	2187 ± 53
10 > TPM ≥ 1	7254 ± 281	8771	5568	4586	4967 ± 229
TPM ≥ 10	5112 ± 638	3203	1959	1580	1307 + 88
	**Junctions**				
TPM < 0.1	24302 ± 3980	55356	54227	63026	107673 ± 1855
0.5 > TPM ≥0.1	32095 ± 3980	70978	58738	68798	95009 ± 1158
1 > TPM ≥ 0.5	21823 ± 3106	45310	28151	38621	52799 ± 1620
10 > TPM ≥ 1	144588 ± 18131	178510	63872	101345	120282 ± 7253
TPM ≥ 10	154833 ± 23376	66807	26405	40517	29891 ± 2066

Supplementary Table S3. The value of slopes from the significant results of the Mann-Kendall Trend test (p-value < 0.05).

	**Heart (DCM) Sample 1**	**Heart (DCM) Sample 2**	**Heart (DCM) Sample 3**	**Heart (DCM) Sample 4**	**Adipose (pre-treatment)**	**Adipose (post-treatment)**
	Genes (In at least one subsample)					
TPM < 0.1	304.67	310.67	291.33	325.0	165.33	No trend
0.5 > TPM ≥ 0.1	41.67	66.33	57.33	67.33	No trend	No trend
1 > TPM ≥ 0.5	11.0	15.33	27.33	27.33	29	16.67
10 > TPM ≥ 1	44.0	39.4	38.0	42.33	49	No trend
TPM ≥ 10	25.0	28.0	27.0	32.0	22	4.33

	Genes (In 100 subsamples)					
TPM < 0.1	394.0	379.67	377.0	376.0	294.33	240.33
0.5 > TPM ≥ 0.1	236.67	245.33	237.67	232.0	294.67	224.67
1 > TPM ≥ 0.5	82.0	90.33	89.33	87.67	95	86.67
10 > TPM ≥ 1	129.33	132.0	136.0	139.0	195.33	123.67
TPM ≥ 10	40.67	43.33	45.0	51.0	47.33	39
	Junctions (In at least one subsample)					
TPM < 0.1	2188.33	2141.33	2029.33	2099.67	979	No trend
0.5 > TPM ≥ 0.1	No trend	No trend	752.67	801.67	No trend	No trend
1 > TPM ≥ 0.5	No trend	No trend	No trend	No trend	823.33	378
10 > TPM ≥ 1	162.0	150.67	109.0	216.0	No trend	No trend
TPM ≥ 10	158.33	173.33	177.0	191.33	251	No trend
	Junctions (In 100 subsamples)					
TPM < 0.1	1933.0	1925.4	1834.0	1758.0	1384.33	937
0.5 > TPM ≥ 0.1	1865.0	1851.67	1817.0	1770.33	1851.33	1238
1 > TPM ≥ 0.5	625.0	731.0	731.33	682.33	884.33	1365
10 > TPM ≥ 1	932.0	1059.0	1059.0	1052.0	1989.67	902.67
TPM ≥ 10	204.0	228.0	223.0	230.33	470.67	263

Supplementary Table S4. The increase of the mean number and the percentage of genes and junctions involved in alternative splicing with increasing sequencing depth in the Adipose (pre-treatment) dataset. Column '50 M' indicates the mean number and the percentage of

genes/junctions detected in the samples with a sequencing depth of less than 50 M reads. The column '300M' indicates the mean number and the percentage of genes/junctions detected in the samples with the highest sequencing depth of 300M reads. Grey shading indicates an increase of less than 1% of new detections.

TPM	50M	50-100	100-150	150-200	200-250	250-300	300M	Total # of genes/ junctions
	**Genes (In at least one subsample)**							
< 0.1	2005 (16.9%)	+635 (+5.4%)	+324 (+2.7%)	+136 (+1.1%)	+36 (+0.3%)	-24 (-0.2%)	3112 (26.2%)	11863
[0.1, 0.5)	1970 (36.5%%)	+460 (+8.5%%)	+176 (+3.2%%)	+65 (+1.2%%)	-7 (-0.1%%)	-14 (-0.3%)	2650 (49.1%%)	5394
[0.5, 1)	1286 (54.0%%)	+185 (+7.8%)	62 (+2.6%)	15 (+0.6%)	8 (+0.3%)	-2 (-0.08%)	1554 (65.3%%)	2380
[1, 10)	3765 (67.6%)	+202 (+3.6%)	+94 (+1.7%)	+40 (+0.7%)	+13 (+0.2%)	+8 (+0.1%)	4122 (74.0%)	5568
≥ 10	1381 (70.5%%)	94 (+4.7%)	39 (+2.0%)	18 (+0.9%)	9 (+0.5%)	5 (+0.3%)	1546 (78.9%)	1959
	**Genes (In 100 subsamples)**							
< 0.1	858 (7.2%)	495 (+4.2%)	346 (+2.9%)	273 (+2.3%)	264 (+2.2%)	248 (+2.1%)	2484 (20.9%)	11863
[0.1, 0.5)	703 (13.0%)	569 (+10.5%)	384 (+7.1%)	279 (+5.2%)	221 (+4.1%)	167 (+3.1%)	2323 (43.0%)	5394
[0.5, 1)	620 (26.1%)	383 (+16.1%)	171 (+7.2%)	122 (+5.1%)	75 (+3.2%)	66 (+2.8%)	1437 (60.4%)	2380
[1, 10)	2591 (46.5%)	709 (+12.7%)	310 (+5.6%)	159 (+2.9%)	117 (+2.1%)	89 (+1.6%)	3975 (71.4%)	5568
≥ 10	1225 (62.5%)	137 (+7.0%)	78 (+4.0%)	40 (+2.0%)	24 (+1.2%)	21 (+1.1%)	1525 (77.8%)	1959
	Junctions (In at least one subsample)							
< 0.1	7472 (13.8%)	+3345 (+6.2%)	+1832 (+3.4%)	+928 (+1.5%)	+276 (+0.5%)	-8 (-0.01%)	13746 (25.3%)	54227
[0.1, 0.5)	8810 (15.0%)	+4448 (+7.6%)	+2028 (+3.5%)	+634 (+1.1%)	+90 (+0.2%)	-130 (-0.2%)	15880 (27.0%)	58738
[0.5, 1)	6446 (22.9%)	+2215 (+7.9%)	+883 (+3.1%)	+228 (+0.8%)	-89 (-0.3%)	-85 (+0.3%)	9598 (34.1%)	28151
[1, 10)	19043 (29.8%)	+3399 (+5.3%)	+956 (+1.5%)	+80 (+0.1%)	-135 (-0.2%)	-22 (-0.03%)	23321 (36.5%)	63872
≥ 10	5539 (21.0%)	+782 (+3.0%)	+370 (+1.4%)	+250 (+0.9%)	+133 (+0.5%)	+157 (+0.6%)	7231 (27.4%)	26405

	Junctions (In 100 subsamples)							
< 0.1	2502 (4.6%)	+1831 (+3.4%)	+1520 (+2.8%)	+1317 (+2.4%)	+1316 (+2.4%)	+1376 (+2.5%)	9862 (18.2%)	54227
[0.1, 0.5)	2117 (2.6%)	+2507 (+4.2%)	+2170 (+3.7%)	+1724 (+2.9%)	+1660 (+2.8%)	+1700 (+2.9%)	11878 (20.2%)	58738
[0.5, 1)	2018 (7.2%)	+1789 (+6.4%)	+1280 (+4.5%)	+957 (+3.4%)	+841 (+3.0%)	+790 (+2.8%)	7675 (27.3%)	28151
[1, 10)	9075 (14.2%)	+4216 (+6.6%)	+2661 (+4.2%)	+1812 (+2.8%)	+1496 (+2.3%)	+1369 (+2.1%)	20629 (32.3%)	63872
≥ 10	4089 (15.5%)	+953 (+3.6%)	+623 (+2.4%)	+430 (+1.6%)	+359 (+1.4%)	+352 (+1.3%)	6806 (25.8%)	26405

Supplementary Table S5. The increase of the mean number and the percentage of genes and junctions involved in alternative splicing with increasing sequencing depth in the Adipose (post-treatment) dataset. Column '50 M' indicates the mean number and the percentage of genes/junctions detected in the samples with a sequencing depth of less than 50 M reads. The column '300M' indicates the mean number and the percentage of genes/junctions detected in the samples with the highest sequencing depth of 300M reads. Grey shading indicates an increase of less than 1% of new detections.

TPM	50M	50-100	100-150	150-200	200-250	250-300	300M	
	Genes (In at least one subsample)							
< 0.1	2066 (17.2%)	513 (+4.3%)	194 (+1.6%)	59 (+0.5%)	-3 (-0.02%)	-38 (-0.3%)	2791 (23.2%)	12010
[0.1, 0.5)	1963 (37.4%)	377 (+7.2%)	69 (+1.3%)	12 (+0.2%)	-9 (-0.2%)	-29 (-0.6%)	2383 (45.4%)	5249
[0.5, 1)	1150 (55.2%)	111 (+5.0%)	4 (+0.2%)	-14 (-0.6%)	-5 (-0.2%)	-17 (-0.8%)	1229 (55.8%)	2204
[1, 10)	2900 (63.2%)	75 (+1.6%)	6 (+0.1%)	-16 (-0.3%)	-14 (-0.3%)	-25 (-0.5%)	2926 (63.8%)	4586
≥ 10	975 (61.7%)	46 (+2.9%)	13 (+0.8%)	-1 (-0.06%)	1 (+0.06%)	1 (+0.06%)	1035 (65.5%)	1580
	Genes (In 100 subsamples)							
< 0.1	766 (6.4%)	+444 (+3.7%)	+310 (+2.6%)	+232 (+1.9%)	+179 (+1.5%)	+144 (+1.2%)	2075 (17.3%)	12010
[0.1, 0.5)	653 (12.4%)	+487 (+9.3%)	+328 (+6.2%)	+208 (+4.0%)	+138 (+2.6%)	+134 (+2.6%)	1948 (37.1%)	5249
[0.5, 1)	510 (23.1%)	+255 (+11.6%)	+135 (+6.1%)	+88 (+4.0%)	+51 (+2.3%)	+39 (+1.8%)	1078 (48.9%)	2204
[1, 10)	1892 (41.3%)	+438 (+9.6%)	+190 (+4.1%)	+102 (+2.2%)	+79 (+1.7%)	+50 (+1.1%)	2751 (60.0%)	4586

≥ 10	780 (49.4%)	+106 (+6.7%)	+44 (+2.8%)	+39 (+2.5%)	+17 (+1.1%)	+20 (+1.3%)	1006 (63.7%)	1580
				Junctions (In at least one subsample)				
< 0.1	6889 (10.9%)	+2454 (+3.9%)	+975 (+1.5%)	+277 (+0.4%)	−64 (−0.1%)	−182 (−0.3%)	10349 (16.4%)	63026
[0.1, 0.5)	8221 (11.9%)	2959 (+4.3%)	847 (+1.2%)	240 (+0.3%)	−251 (−0.2%)	−320 (−0.5%)	11794 (17.1%)	68798
[0.5, 1)	5510 (13.3%)	1196 (+3.1%)	192 (+0.5%)	−119 (−0.3%)	−117 (−0.3%)	−149 (−0.4%)	6153 (15.9%)	38621
[1, 10)	13327 (13.2%)	1071 (+1.1%)	−17 (−0.02%)	−218 (−0.2%)	−285 (−0.3%)	−177 (−0.2%)	13701 (13.5%)	101345
≥ 10	3918 (9.7%)	450 (+1.1%)	172 (+0.4%)	44 (+0.1%)	−13 (−0.03%)	−5 (−0.01%)	4566 (11.3%)	40517
				Junctions (In 100 subsamples)				
< 0.1	2061 (3.3%)	+1438 (+2.3%)	+1138 (+1.8%)	+937 (+1.5%)	+676 (+1.1%)	+679 (+1.1%)	6930 (11.0%)	63026
[0.1, 0.5)	1934 (2.8%)	+1949 (+2.8%)	+1505 (+2.2%)	+1238 (+1.8%)	+871 (+1.3%)	+805 (+1.2%)	8302 (12.1%)	68798
[0.5, 1)	1605 (4.2%)	+1161 (+3.0%)	+750 (+1.9%)	+540 (+1.4%)	+415 (+1.1%)	+362 (+0.9%)	4822 (12.5%)	38621
[1, 10)	6301 (6.2%)	+2317 (+2.3%)	+1188 (+1.2%)	+882 (+0.9%)	+638 (+0.6%)	+560 (+0.6%)	11886 (11.7%)	101345
≥ 10	2491 (6.1%)	+447 (+1.1%)	+301 (+0.7%)	+263 (+0.6%)	+184 (+0.5%)	+197 (+0.5%)	3883 (9.6%)	40517

Supplementary Table S6. The increase of the mean number and the percentage of genes and junctions involved in alternative splicing with increasing sequencing depth in the Hypothalamus dataset. Column '50 M' indicates the mean number and the percentage of genes/junctions detected in the samples with a sequencing depth of less than 50 M reads. The column '200M' indicates the mean number and the percentage of genes/junctions detected in the samples with the highest sequencing depth of 300M reads. Grey shading indicates an increase of less than 1% of new detections.

TPM	50M	50-100	100-150	150-200	200M	Total # of genes/ junctions
			Genes (In at least one subsample)			
< 0.1	1925 (18.7%)	517 (+5.0%)	250 (+2.4%)	10 (+0.1%)	2702 (26.2%)	10303
[0.1, 0.5)	1509 (24.4%)	454 (+7.3%)	184 (+3.0%)	20 (+0.3%)	2167 (35.1%)	6179
[0.5, 1)	1239 (43.9%)	220 (+7.8%)	82 (+2.9%)	15 (+0.5%)	1556 (55.1%)	2825

[1, 10)	5929 (67.6%)	431 (+4.9%)	155 (+1.8%)	28 (+0.3%)	6543 (74.6%)	8771
≥ 10	2531 (79.0%)	85 (+2.7%)	26 (+0.8%)	26 (+0.8%)	2668 (83.3%)	3203
	Genes (In 100 subsamples)					
< 0.1	1074 (10.4%)	+576 (+5.6%)	+383 (+3.7%)	+421 (+4.1%)	2454 (23.8%)	10303
[0.1, 0.5)	604 (9.8%)	+566 (+9.2%)	+388 (+6.3%)	+388 (+6.3%)	1946 (31.5%)	6179
[0.5, 1)	592 (21.0%)	+435 (+15.4%)	+242 (+8.6%)	+194 (+6.9%)	1463 (51.8%)	2825
[1, 10)	4222 (48.1%)	+1340 (+15.3%)	+501 (+5.7%)	+336 (+3.8%)	6399 (80.0%)	8771
≥ 10	2315 (72.3%)	+1544 (+6.4%)	+573 (+2.2%)	+395 (+1.8%)	2650 (82.7%)	3203
	Junctions (In at least one subsample)					
< 0.1	8661 (15.6%)	+2939 (+5.3%)	+1275 (+2.3%)	+57 (+0.1%)	12932 (23.4%)	55356
[0.1, 0.5)	7869 (11.1%)	+3634 (+5.1%)	+1638 (+2.3%)	−19 (−0.03%)	13122 (18.5%)	70978
[0.5, 1)	7217 (15.9%)	+2392 (+5.3%)	+836 (+1.8%)	−36 (−0.08%)	10409 (23.0%)	45310
[1, 10)	38921 (21.8%)	+7237 (+4.1%)	+1840 (+1.0%)	+196 (+0.1%)	48194 (27.0%)	178510
≥ 10	15363 (23.0%)	1795 (+2.7%)	915 (+1.4%)	466 (+0.7%)	18539 (27.8%)	66807
	Junctions (In 100 subsamples)					
< 0.1	3642 (6.6%)	+2774 (+5.0%)	+2177 (+3.9%)	+2601 +(4.7%)	11194 (20.2%)	55356
[0.1, 0.5)	2177 (3.1%)	+2842 (+4.0%)	+2734 (+3.9%)	+3069 (+4.3%)	10822 (15.2%)	70978
[0.5, 1)	2221 (4.9%)	+2717 (+6.0%)	+2065 (+4.6%)	+2087 (+4.6%)	9090 (20.1%)	45310
[1, 10)	18321 (10.3%)	+11842 (+6.6%)	+7313 (+4.1%)	+6926 (+3.9%)	44402 (24.9%)	178510
≥ 10	10238 (5.4%)	+15422 (+5.4%)	+9408 (+3.1%)	+8755 (+2.7%)	17742 (26.6%)	66807

Supplementary Table S7. The results of NEASE with KEGG pathways for the deep-sequenced data sets

KEGG Pathway	P-adjusted
**Adipose (pre-treatment)**	
Citrate cycle (TCA cycle)	0.0052
Propanoate metabolism	0.0052
Apoptosis	0.0059
PI3K-Akt signaling pathway	0.0061
p53 signaling pathway	0.0078
Small cell lung cancer	0.0087
Gastric cancer	0.0107
Pyruvate metabolism	0.011
Ferroptosis	0.014
Ras signaling pathway	0.016
Antigen processing and presentation	0.018
Prostate cancer	0.02
VEGF signaling pathway	0.021
Hepatocellular carcinoma	0.025
Vasopressin-regulated water reabsorption	0.03
Thyroid hormone signaling pathway	0.038
Non-small cell lung cancer	0.04
Valine, leucine and isoleucine degradation	0.042
Hepatitis B	0.042
Fatty acid degradation	0.043
Acute myeloid leukemia	0.044
**Adipose (post-treatment)**	
SNARE interactions in vesicular transport	0.0056
NOD-like receptor signaling pathway	0.0059

Herpes simplex infection	0.0103
Epstein-Barr virus infection	0.0103
TNF signaling pathway	0.0103
Toll-like receptor signaling pathway	0.021
Measles	0.024
Antigen processing and presentation	0.026
Endocytosis	0.028
mTOR signaling pathway	0.031
Proteasome	0.031
**Hypothalamus**	
Prion diseases	0.0048
Phagosome	0.0055
Gap junction	0.007
Oxidative phosphorylation	0.0092
Adherens junction	0.023
Glutamatergic synapse	0.023
Insulin secretion	0.024
Propanoate metabolism	0.025
Fatty acid degradation	0.025
Fructose and mannose metabolism	0.025
Leishmaniasis	0.026
Pancreatic secretion	0.026
Glycolysis / Gluconeogenesis	0.026
Hippo signaling pathway	0.026
Valine, leucine and isoleucine degradation	0.026
Protein processing in endoplasmic reticulum	0.027
Oocyte meiosis	0.027

Collecting duct acid secretion	0.032
Carbohydrate digestion and absorption	0.044
cAMP signaling pathway -	0.046
Human T-cell leukemia virus 1 infection	0.048

1. Lee,K., Yu,D., Hyung,D., Cho,S.Y. and Park,C. (2022) ASpediaFI: Functional Interaction Analysis of Alternative Splicing Events. *Genomics Proteomics Bioinformatics*, **20**, 466–482.

2. Fahmi,N.A., Nassereddeen,H., Chang,J., Park,M., Yeh,H., Sun,J., Fan,D., Yong,J. and Zhang,W. (2021) AS-Quant: Detection and Visualization of Alternative Splicing Events with RNA-seq Data. *Int. J. Mol. Sci.*, **22**.

3. Qi,H., Guo,X., Wang,T. and Zhang,Z. (2022) ASTool: An Easy-to-Use Tool to Accurately Identify Alternative Splicing Events from Plant RNA-Seq Data. *Int. J. Mol. Sci.*, **23**.

4. Li,H.-D., Funk,C.C. and Price,N.D. (2020) iREAD: a tool for intron retention detection from RNA-seq data. *BMC Genomics*, **21**, 128.

5. Kahles,A., Ong,C.S., Zhong,Y. and Rätsch,G. (2016) SplAdder: identification, quantification and testing of alternative splicing events from RNA-Seq data. *Bioinformatics*, **32**, 1840–1847.

6. Alqassem,I., Sonthalia,Y., Klitzke-Feser,E., Shim,H. and Canzar,S. (2021) McSplicer: a probabilistic model for estimating splice site usage from RNA-seq data. *Bioinformatics*, **37**, 2004–2011.

7. Zhou,J., Ma,S., Wang,D., Zeng,J. and Jiang,T. (2018) FreePSI: an alignment-free approach to estimating exon-inclusion ratios without a reference transcriptome. *Nucleic Acids Res.*, **46**, e11.

8. Trincado,J.L., Entizne,J.C., Hysenaj,G., Singh,B., Skalic,M., Elliott,D.J. and Eyras,E. (2018) SUPPA2: fast, accurate, and uncertainty-aware differential splicing analysis across multiple conditions. *Genome Biol.*, **19**, 40.

9. Ma,X. and Zhang,X. (2013) NURD: an implementation of a new method to estimate isoform expression from non-uniform RNA-seq data. *BMC Bioinformatics*, **14**, 220.

10. Trapnell,C., Hendrickson,D.G., Sauvageau,M., Goff,L., Rinn,J.L. and Pachter,L. (2013) Differential analysis of gene regulation at transcript resolution with RNA-seq. *Nat. Biotechnol.*, **31**, 46–53.

11. Wu,W., Zong,J., Wei,N., Cheng,J., Zhou,X., Cheng,Y., Chen,D., Guo,Q., Zhang,B. and Feng,Y. (2018) CASH: a constructing comprehensive splice site method for detecting alternative splicing events. *Brief. Bioinform.*, **19**, 905–917.

12. Ferrer-Bonsoms,J.A., Gimeno,M., Olaverri,D., Sacristan,P., Lobato,C., Castilla,C., Carazo,F. and Rubio,A. (2022) EventPointer 3.0: flexible and accurate splicing analysis that includes studying the differential usage of protein-domains. *NAR Genom Bioinform*, **4**,

lqac067.

13. Manz,Q., Tsoy,O., Fenn,A., Baumbach,J., Völker,U., List,M. and Kacprowski,T. (2021) ASimulatoR: splice-aware RNA-Seq data simulation. *Bioinformatics*, **37**, 3008–3010.

14. Fenn,A., Tsoy,O., Faro,T., Rößler,F.L.M., Dietrich,A., Kersting,J., Louadi,Z., Lio,C.T., Völker,U., Baumbach,J., *et al.* (2023) Alternative splicing analysis benchmark with DICAST. *NAR Genom Bioinform*, **5**, lqad044.
